# Colloidal Aziridinium Lead Bromide Quantum Dots

**DOI:** 10.1021/acsnano.3c11579

**Published:** 2024-02-06

**Authors:** Maryna I. Bodnarchuk, Leon G. Feld, Chenglian Zhu, Simon C. Boehme, Federica Bertolotti, Jonathan Avaro, Marcel Aebli, Showkat Hassan Mir, Norberto Masciocchi, Rolf Erni, Sudip Chakraborty, Antonietta Guagliardi, Gabriele Rainò, Maksym V. Kovalenko

**Affiliations:** † Laboratory for Thin Films and Photovoltaics, Empa, Swiss Federal Laboratories for Materials Science and Technology, Dübendorf 8600, Switzerland; ‡ Institute of Inorganic Chemistry, Department of Chemistry and Applied Biosciences, ETH Zürich, Zürich 8093, Switzerland; § SKKU Institute of Energy Science and Technology (SIEST), Sungkyunkwan University, Suwon 16419, South Korea; ⧫ Electron Microscopy Center, Empa, Swiss Federal Laboratories for Materials Science and Technology, Dübendorf 8600, Switzerland; ◊ Centre for X-ray Analytics & Laboratory for Biomimetic Membranes and Textiles, Empa, Swiss Federal Laboratories for Materials Science and Technology, St. Gallen 9014, Switzerland; # Department of Science and High Technology and To.Sca.Lab., University of Insubria, via Valleggio 11, Como 22100, Italy; ○ Istituto di Cristallografia and To.Sca.Lab, Consiglio Nazionale delle Ricerche, via Valleggio 11, Como 22100, Italy; ☆ Materials Theory for Energy Scavenging (MATES) Lab, Harish-Chandra Research Institute (HRI) Allahabad, A C.I. of Homi Bhabha National Institute (HBNI), Chhatnag Road, Jhunsi, Prayagraj (Allahabad) 211019, India

**Keywords:** nanocrystals, quantum dots, aziridinium, perovskite, ligands, photoluminescence

## Abstract

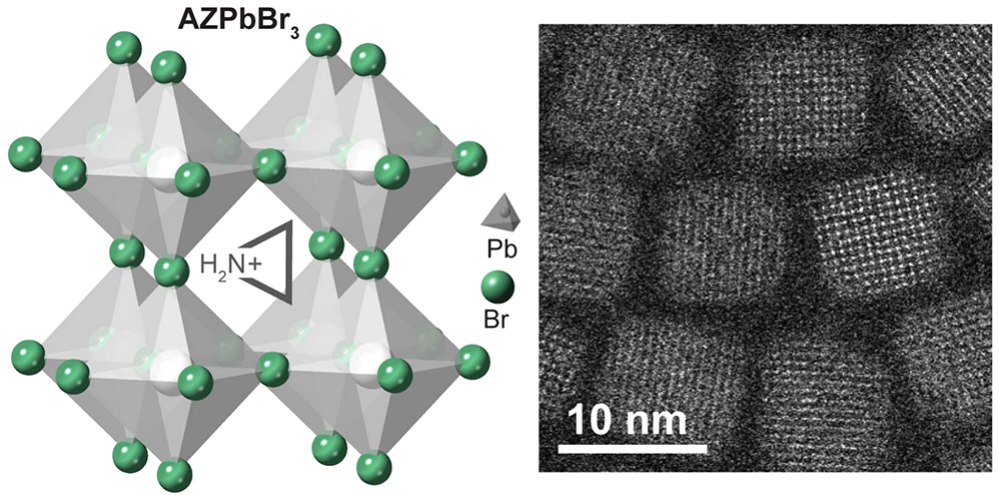

The compositional engineering of lead-halide perovskite nanocrystals (NCs) via the A-site cation represents a lever to fine-tune their structural and electronic properties. However, the presently available chemical space remains minimal since, thus far, only three A-site cations have been reported to favor the formation of stable lead-halide perovskite NCs, i.e., Cs^+^, formamidinium (FA), and methylammonium (MA). Inspired by recent reports on bulk single crystals with aziridinium (AZ) as the A-site cation, we present a facile colloidal synthesis of AZPbBr_3_ NCs with a narrow size distribution and size tunability down to 4 nm, producing quantum dots (QDs) in the regime of strong quantum confinement. NMR and Raman spectroscopies confirm the stabilization of the AZ cations in the locally distorted cubic structure. AZPbBr_3_ QDs exhibit bright photoluminescence with quantum efficiencies of up to 80%. Stabilized with cationic and zwitterionic capping ligands, single AZPbBr_3_ QDs exhibit stable single-photon emission, which is another essential attribute of QDs. In particular, didodecyldimethylammonium bromide and 2-octyldodecyl-phosphoethanolamine ligands afford AZPbBr_3_ QDs with high spectral stability at both room and cryogenic temperatures, reduced blinking with a characteristic ON fraction larger than 85%, and high single-photon purity (g^(2)^(0) = 0.1), all comparable to the best-reported values for MAPbBr_3_ and FAPbBr_3_ QDs of the same size.

The past decade has seen the discovery and the rapid development of colloidal lead halide perovskite nanocrystals (NCs) of APbX_3_ stoichiometry, where A represents the Cs cation or an organic cation, and X indicates a halogen anion.^1−3^ These materials have captured broad interest due to their straightforward synthesis and outstanding optical properties, foremost narrowband photoluminescence (PL) with near-unity quantum yield (QY) and wide spectral tunability (410–750 nm), high absorption coefficients and, at cryogenic temperatures, high radiative rates, and long excitonic coherence times.^4−7^ A rather stringent structural requirement for forming the perovskite lattice (i.e., a three-dimensional (3D) network of corner-shared lead-halide octahedra), usually expressed via the Goldschmidt tolerance factor, is the insertion of A-site cations of an appropriate size. Cs is the only sufficiently large inorganic cation, whereas suitably small and yet chemically robust organic cations include methylammonium (MA) and formamidinium (FA), both extensively used in perovskites as optoelectronic materials. Judicious choices of these three ions in either single- or mixed-cation compositions enable fine-tuning of electronic properties via, e.g., the octahedral tilt angles, as well as improving materials phase stability and chemical durability in bulk, thin-film, and nanocrystalline forms.^8−12^ Further expanding the thus-far-limited choice for the A-site cation may unlock additional opportunities in the precision engineering of the structural and electronic properties of lead-halide perovskites.

Very recently, the aziridinium cation [(CH_2_)_2_NH_2_^+^, labeled hereafter as AZ], a highly reactive and labile triangular heterocycle, was reported to form stable AZPbX_3_ compounds.^13−15^ Seemingly in contradiction to the latter, computational assessments of the ring opening within the formed perovskite A-site voids had yielded an enthalpy difference of 0.7 eV per formula unit (f.u.) in favor of ring opening in AZPbI_3_.^16^ However, this value is smaller than the computed and experimental ring-strain energies of ca. 27 kcal/mol (1.16 eV/f.u.),^17,18^ suggesting an overall stabilizing effect by the Pb-halide cage as a “counter-strain” due to the A-site being too small for the opened ring. Furthermore, several favorable formation conditions exist. First, the size of the three-membered AZ ring cation is similar to that of the commonly used MA cation, and the Goldschmidt tolerance factor for AZPbBr_3_ is 0.95 (the used ionic radii are *r*_Br_ = 196 pm, *r*_Pb_ = 119 pm, *r*_AZ_ = 227 pm),^19^ well within the perovskite formability limits (Figure 1a). Second, the thermodynamic stability of AZPbX_3_ (as well as that of Sn analogues) has been anticipated in first-principles calculations focused on the ionization energy of the A-site as a stability predictor.^19−22^ Since the AZ moiety, in its neutral form, exhibits a lower ionization energy than (neutral) MA, with a value closely resembling that of atomic Cs, AZPbX_3_ phases were expected to be more stable than MAPbX_3_ with thermochemical calculations further supporting the formation of stable perovskite phases. A remaining concern is the reactivity of AZ (polymerization, nucleophilic attack on the ring, etc.), which also breaks its cyclic nature. However, here again, the perovskite cage apparently guards the AZ cations, as seen from the air stability of the reported AZPbX_3_ compounds.^13−15^ Additionally, strong steric requirements provided by the size and shape of the “reaction cavity” within the crystal lattice may increase the actual activation energy for any thermodynamically allowed transformation to a threshold not easily achievable if no additional power is provided (in the form of heat or light). Indeed, when the space surrounding the molecules becomes restricted, their reactivity and related behavior can be significantly altered.^23^

**Figure 1:**
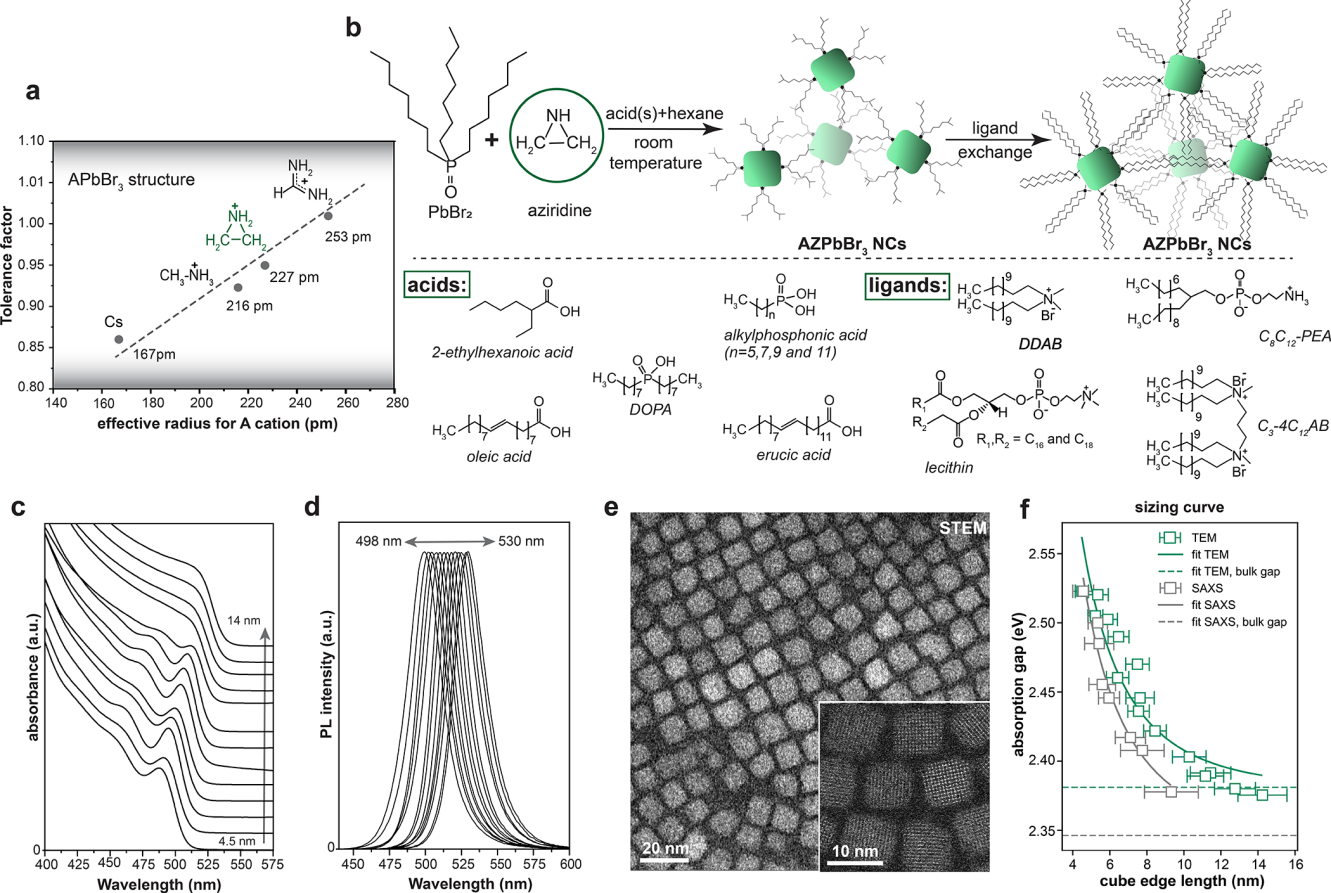
(a) Calculated Goldschmidt tolerance factors for different cations (cesium, methylammonium, aziridinium, and formamidinium) in the APbBr_3_ perovskite lattice. (b) Top panel: a reaction scheme; bottom panel: an overview of carboxylic and phosphonic acids as well as ligands used in the synthesis. (c) Optical absorption and (**d**) PL spectra of purified AZPbBr_3_ NCs ranging from 4.5 to 14 nm in size (for visualization purposes, a cumulative vertical offset was applied to each subsequent spectra). (e) A high-angle annular dark field-scanning transmission electron microscopy (HAADF-STEM) image of purified 8 nm NCs with a high-resolution HAADF-STEM image of few single NC in the inset. (f) Size-dependent (absorption) band gap in AZPbBr_3_ NCs for sizes obtained via TEM (green) and SAXS (gray); the experimental data sets (open squares, with error bars denoting the standard deviation) were fitted by a semiempirical sizing curve (solid line), with the bulk band gap (dashed lines) as one of the fit parameters.

In this work, we explored the prospects of AZ-based perovskite NCs as a potentially powerful addition to the family of highly luminescent MA/FA/Cs lead halide NCs. We focused on the AZPbBr_3_ composition and devised the synthesis of monodisperse and size-tunable NCs in strong and weak quantum-confinement regimes, e.g., QDs. The preparation of AZPbBr_3_ NCs leveraged the recently reported room-temperature synthesis platform based on PbBr_2_/trioctylphosphine oxide (TOPO) molecular adducts as the precursor,^24^ wherein the formation of NCs is precisely adjustable and traceable in situ, due to slower reaction kinetics, compared to more traditional hot-injection or solvent-assisted reprecipitation methods. Furthermore, this PbBr_2_/TOPO synthesis benefits from the decoupling of the NC formation and subsequent coating with the capping ligand of choice before the purification and isolation of NCs. We tested several state-of-the-art capping ligands, cationic and zwitterionic, and found that didodecyldimethylammonium bromide (DDAB) and 2-octyldodecyl-phosphoethanolamine (C_8_C_12_–PEA) ligands render AZPbBr_3_ NCs robust for both ensemble and single-particle studies. Their photostability, high ON-fraction (>85%) in blinking studies, and high single-photon purity (*g*^(2)^(0) as low as 0.1) render AZPbBr_3_ NCs comparable to the best reported MAPbBr_3_ and FAPbBr_3_ NCs.^25^

## Results and Discussion

### Synthesis of AZPbBr_3_ NCs

The room-temperature synthesis of AZPbBr_3_ QDs proceeds through rapid injection of an aziridine solution in chloroform or dibromomethane (see further details in Figure 1b and the Supporting Information) into a precursor solution comprising PbBr_2_/TOPO adduct, diisooctylphosphinic (DOPA), and alk(en)ylcarboxylic acids (2-ethylhexanoic acid, oleic acid, or erucic acid) in hexane, with the optional addition of alkylphosphonic acids (for obtaining the smallest NCs; see the subsequent discussion). We note that AZ cations cannot be formed ex situ for use as a stable precursor, due to their high ring instability in common solvents. Instead, AZ cations form in situ, because of the high acidity of the PbBr_2_/TOPO precursor solution (aziridine:DOPA:carboxylic acids molar ratios = 1:8.5:17). TOPO, DOPA, and alkyl carboxylic acids are known as weakly binding ligands for perovskite NCs.^24^ They can be readily displaced by more strongly binding alternatives such as didodecyldimethylammonium bromide (DDAB),^26,27^ custom-engineered zwitterionic phospholipid ligand [2-octyldodecylphosphoethanolamine (C_8_C_12_–PEA)],^25^ or the commercially available natural phospholipid lecithin,^28^ followed by purification and isolation steps. This procedure yields stable colloids of highly monodispersed cuboidal AZPbBr_3_ NCs exhibiting bright green emission and high stability over long-term storage in air (Figure S1).

The size of NCs was adjusted between 4.5 and 14 nm in diameter, resulting in tunable absorption and PL, with PL peaks in the range from 498 to 530 nm (see Figures 1c and 1d, as well as Figures S2 and S3 and Table S1), by manipulating the reaction time (the time delay between the injection of the aziridine solution and the injection of the ligand solution was typically 10–240 s) as well as by introducing various amounts of alkylphosphonic acids (hexyl-, octyl-, decyl-, or dodecylphosphonic acid) into the PbBr_2_/TOPO precursor solution. Phosphonic acids slow the reaction kinetics, facilitating the preparation of strongly confined AZPbBr_3_ NCs (down to 4.5 nm, Figure S2d). Conversely, utilizing mesitylene as a reaction solvent and a longer reaction time yields NCs larger than 10 nm, with PL peaks from 525 to 530 nm. The overall dilution of precursors does not significantly alter the PL peak of AZPbBr_3_ (Figure S2c), unlike in the synthesis of CsPbBr_3_ NCs.^24^ Importantly, a narrow size dispersion of AZPbBr_3_ NCs can be reached only under a high Pb-precursor excess (aziridine:Pb molar ratio = 1:4). The minute-scale formation kinetics of AZPbBr_3_ NCs allow in situ optical monitoring with ultraviolet-visible light (UV-vis) absorption, as exemplified for 6.5 nm samples (Figure S4).

DDAB-capped AZPbBr_3_ NCs are sharp cuboids, in agreement with the known tendency of cationic ligands to stabilize the set of (100) facets.^26,29,30^ C_8_C_12_–PEA-coated NCs are rather truncated, presumably due to surface reconstruction or etching (see Figure S3 and Table S1). Lower colloidal robustness was observed when employing the recently reported dicationic ligands (propanediyl-1,3-*N*,*N*-bis(didodecylmethylammonium bromide), C_3_-4C_12_AB)^31^ or lecithin. The AZPbBr_3_ NCs capped with DDAB or C_8_C_12_-PEA exhibit an average PL QY of 80% ± 2% for NC sizes between 8 and 10 nm, and the PL QY increases up to 90% ± 4% for samples prepared with the addition of phosphonic acids (4.5–8 nm).

### Electron Microscopy and SAXS Studies

**Figure 2:**
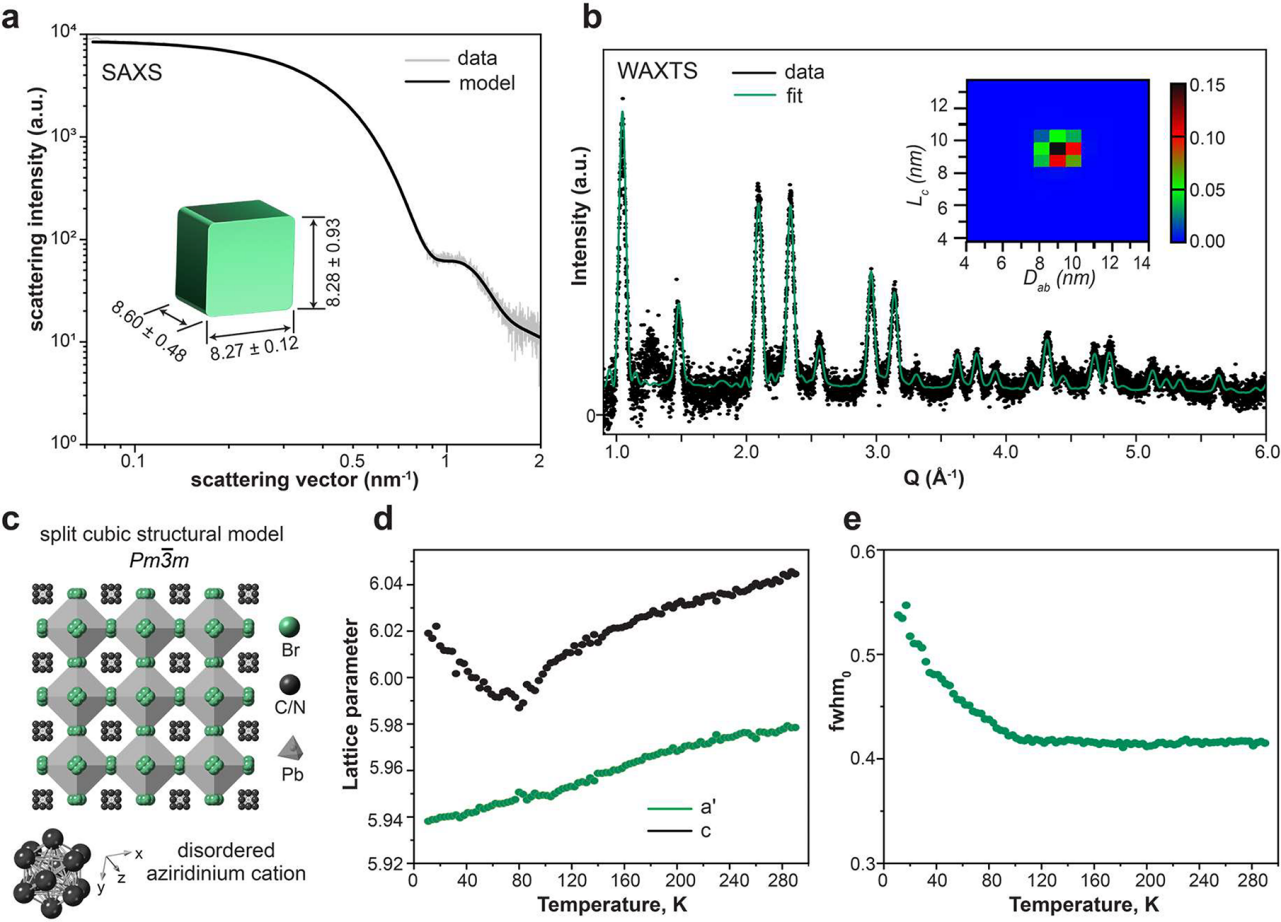
(a) The fit of experimental SAXS data from an AZPbBr_3_ NC colloidal suspension (gray line) via an analytical model (black line) yields cuboids with edge lengths of 8.27 ± 0.12, 8.28 ± 0.93, and 8.60 ± 0.48 nm, respectively. (b) Solvent-subtracted synchrotron WAXTS data (black line) and the best fit (green line) of AZPbBr_3_ NCs using the split cubic model with locally tilted/disordered PbBr_6_ octahedra (for a more extended discussion, see the Supporting Information); the inset shows a 2D map of the refined (number-based) log-normal size distribution function (*D*_ab_: the diameter of the circle of area equivalent to the prisms basal plane and *L*_c_, the height of the prismatic clusters). (c) The split cubic structural model of AZPbBr_3_ NCs (the four Br of each PbBr_6_ octahedra, shown in green, have 1/4 site occupancy factor each, i.e., only one out of four is stochastically present in each site) and the model of AZ cation formed by disordering in 12 symmetry-equivalent orientations (4 equivalent geometrical orientations × 3 “elemental” C–C/N dispositions, H atoms were omitted, similar to bulk AZPbBr_3_). (d) The temperature dependence of the tetragonal unit cell parameters *a*′ = *a*/√2 and *c* for a weakly distorted (ca. 1%) cubic lattice, plotted in the entire 11–290 K range, showing an anomaly below 90 K. (e) The temperature dependence of the fwhm_0_ parameter (the θ-dependence of the peak width is described according to the relation fwhm(θ) = fwhm_0_/cos θ), which suggests additional peak broadening (hidden splitting) below 90 K.

The high-angle annular dark field-STEM (HAADF-STEM) and high-resolution HAADF-STEM images evidence the (100) termination of the DDAB-capped NC surfaces (Figure 1e; synthesis without phosphonic acids, 20 s reaction labeled as “standard” in Table S1). Shape retrieval from small-angle X-ray scattering (SAXS, Figure 2a) yields a slightly prolate cuboid shape (aspect ratio of ca. 1.04) with the lengths of the three NC edges being 8.27 ± 0.12, 8.28 ± 0.93, and 8.60 ± 0.48 nm (that is, substantially isotropic; see Figure S5 and Table S2 in the Supporting Information for SAXS data of an extended NC size series).

### Sizing Curve

Figure 1f presents the size dependence of the band gap energies in AZPbBr_3_, using the NC size determined via either TEM or SAXS, and the band gap estimated from the lowest-energy minimum of the second derivative of the absorbance (see the Supporting Information for details). Such a “sizing curve” is of great practical utility as an express method for obtaining the approximate NC size using only a standard UV-vis spectrometer. Furthermore, the size dependence associates with the basic electronic structure of the underlying bulk material. Within the semiempirical expression derived in Aubert et al.,^32^ (see the Supporting Information for the equation and further details), the functional form of the size dependence for a wide range of semiconductors is given by only three bulk parameters: the bulk band gap (*E*_0_), the reduced mass (μ) of the exciton, and the dielectric constant (ϵ_*∞*_) at the optical frequency (see the Supporting Information for further details). In Figure1f, we have tested the inverse of this idea by fitting our experimental AZPbBr_3_ size-dependent band gap (open squares) with the semiempirical expression (solid line) given by Aubert et al.,^32^ hereby yielding approximate estimates for the thus far still ill-defined electronic parameters for bulk AZPbBr_3_. To limit the parameter space and stabilize the fit, we keep the bulk band gap *E*_0_ and the reduced mass μ as free fit parameters and estimate the dielectric constant ϵ_*∞*_ with the help of DFT calculations (see Table S5 and associated details in the Supporting Information). For the SAXS dataset, the best fit is obtained for the following parameters: *E*_0_ = 2.35 eV (fitted), μ = 0.17 (fitted), and ϵ_*∞*_ = 8.25 (fixed; based on our DFT calculations 13% higher than for CsPbBr_3_^33^), translating into a Bohr diameter *d*_0_ of about 5.0 nm. Similar fit parameters are also obtained for the TEM dataset, albeit with a slightly higher estimate for the bulk band gap. Overall, we conclude that the electron and hole effective masses, dielectric constant, and, thus, the exciton Bohr diameter should be comparable in AZPbBr_3_ and CsPbBr_3_ (see also Tables S4–S6),^32,34^ while the bulk band gap of AZPbBr_3_ appears few tens of millielectronvolts lower than in CsPbBr_3_ (*E*_0_ = 2.38 eV in CsPbBr_3_).^35^ We also note that a previous estimate for the bulk AZPbBr_3_ band gap by Petrosova et al.^13^ found an even lower value (2.27 eV); however, their different definition for the band gap (via the zero-crossing in a Tauc plot) precludes a direct comparison to the values found by us for AZPbBr_3_ and by Mannino et al.^35^ for CsPbBr_3_ (in both cases defined via second derivatives).

### Crystal Structure

The crystal structure of AZPbBr_3_ NCs suspended in cycloheptane was investigated with synchrotron X-ray total scattering methods. Bulk AZPbBr_3_ was previously reported to crystallize in the cubic lattice with a *Pm*3̅*m* space group symmetry and ordered Br atoms with a straight Pb–Br–Pb bond angle of 180°.^13^ However, evidence of local and dynamic symmetry breaking has been found in various lead-halide perovskites that exhibit a long-range cubic structure.^36−40^ We account for local symmetry breaking with a split-cubic perovskite model, for a disordered AZ cation with a cuboctahedral cluster, and for disorder of Br anions into four equivalent positions, as well as a cuboidal NC morphology with the Debye scattering equation. The resulting fit to the wide-angle X-ray total scattering (WAXTS) data is shown in Figure 2b, together with a 2D map of the refined bivariate log-normal size-distribution function. Figure 2c displays the obtained structure, with Pb–Br–Pb bond angles deviating by 13° from the 180° angle expected for an ideal cubic structure. Deviations of similar magnitude are also found in FAPbBr_3_, FAPbI_3_, and FASnI_3_ NCs,^8,12,41−43^ corroborating that AZPbBr_3_ NCs share the locally broken crystal symmetry. Furthermore, AZPbBr_3_ NCs exhibit a slight lattice expansion of ∼0.10%–0.15% with respect to the bulk value, similar to many other nanosized samples of lead-halide perovskites (Table S3).^44−46^ We further uncover signs of a noncubic long-range structure. A small deviation in lattice parameters from a cubic cell (with the *c* axis about 1% larger than the *a* = *b* axis) suggests that the average cell is tetragonal (see Figure S6 and the pertinent discussion in the Supporting Information).

To further understand the crystal symmetry, we performed variable-temperature WAXTS measurements of dried NCs loaded into a glass capillary and collected 93 WAXTS scattering patterns (at 3 K steps, from 11 K to room temperature; see the Supporting Information). Similar to the RT WAXTS data of the solution-phase NCs, also the RT crystal metrics of the dried NCs suggest a distortion from the cubic lattice when analyzed according to the structureless Le Bail method (which avoids the occurrence of the nonrandom orientation distribution function of the NCs and is fully unbiased by errors in the structural model). With decreasing temperature, the crystal unit cell volume monotonously decreases. More importantly, an additional symmetry change is found below 90 K (Figure 2d). From 90 to 11 K, the peak widths progressively increase (Figure 2e), suggesting an additional peak splitting (partially hidden under the broad Bragg peaks), which is consistent with a symmetry-lowering transition to an orthorhombic metric, as observed also in other 3D lead-halide perovskites.

### Computational Study

Having elucidated the experimentally observed crystal structure of the synthesized AZPbBr_3_ NCs, we further consider the crystal stability via a computational approach. The Goldschmidt tolerance factor (*t*)^47^ is used extensively to predict the formation and stability of the perovskite structure. However, some studies suggested that a revision of the Goldschmidt tolerance factors may be required.^48^ Recently, Bartel et al. introduced a different tolerance factor^49^
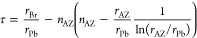
1with *n*_AZ_ representing the oxidation state of the AZ-cation and a value of τ < 4.18 suggests a perovskite structure. We estimated τ = 3.30 for AZPbBr_3_, further supporting the perovskite structure formation (the ionic radii used in the present work were *r*_Br_ = 196 pm, *r*_Pb_ = 119 pm, *r*_AZ_ = 227 pm, and *n*_AZ_ = 1).

To investigate the stability of AZPbBr_3_, we considered the formation reaction equation (CH_2_)_2_NH_2_Br + PbBr_2_ → (CH_2_)_2_NH_2_PbBr_3_ and the corresponding reaction enthalpy:^19^
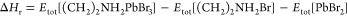
2A negative reaction enthalpy would indicate a stable perovskite structure. To obtain the total energy (*E*_tot_) of the reactant and products, we performed density functional theory (DFT) calculations, employing the PBE exchange-correlation functional with van der Waals (vdW) corrections. We further considered an orthorhombic AZPbBr_3_ crystal structure, the most stable phase at 0 K calculations, in coarse agreement with the lower-than-tetragonal symmetry in our low-temperature X-ray total scattering data (see Supporting Information for further details, including a discussion of the likelihood of polymorphism). We then obtained negative Δ*H*_r_ values of −0.362 eV and −0.373 eV with and without spin–orbit coupling, respectively, affirming the stability of AZPbBr_3_. The respective electronic-structure calculations show a direct band gap of AZPbBr_3_. The conduction band originates from Pb p orbitals, while the valence band is predominantly of Br p character, consistent with other lead halide perovskites (see the Supporting Information for details (Figures S7 and S8, and Tables S7 and S8).

### NMR Study

**Figure 3:**
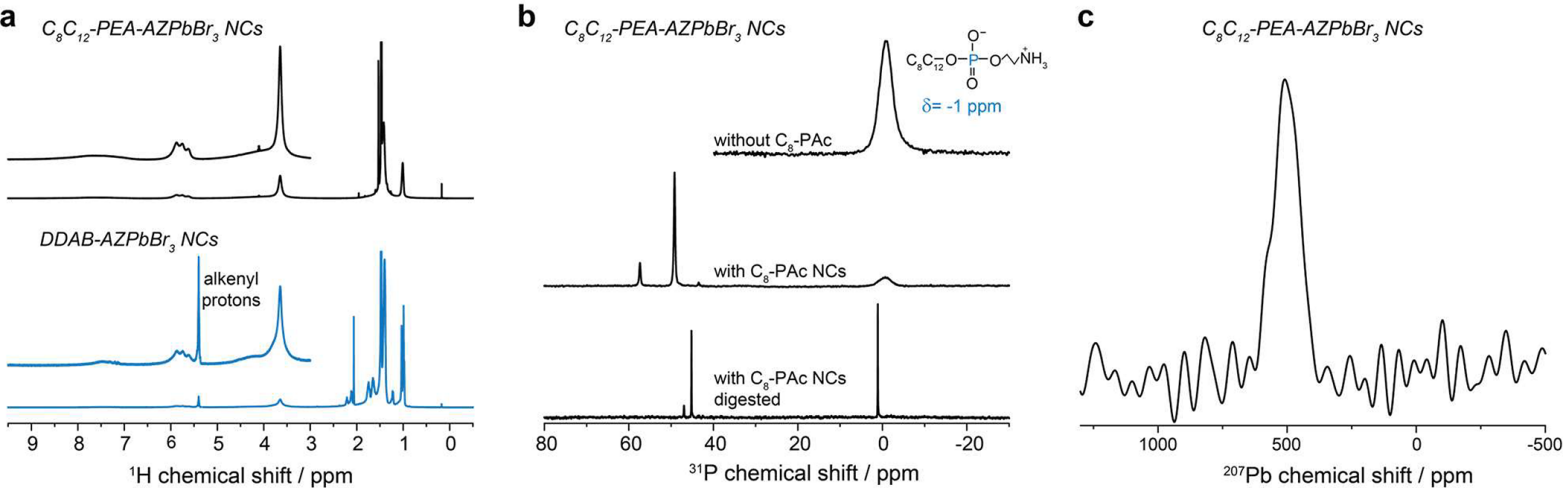
(a) Solution ^1^H NMR spectra of C_8_C_12_–PEA (black line) and DDAB-capped (blue line) AZPbBr_3_ NCs in cyclohexane-*d*_12_. (b) Solution ^31^P NMR spectra of C_8_C_12_–PEA-capped AZPbBr_3_ NCs synthesized without (top) and with (middle) addition of alkylphosphonic acid (for instance, octylphosphonic acid-C_8_PAc) during the synthesis and after their further digestion with DMSO-*d*_6_ (bottom). (c) Solid-state ^207^Pb NMR spectrum of C_8_C_12_–PEA-capped AZPbBr_3_ NCs.

The ligand chemistry of C_8_C_12_–PEA and DDAB-capped AZPbBr_3_ NCs was elucidated with NMR experiments performed in solution and the solid state. ^1^H solution NMR spectra for both C_8_C_12_–PEA and DDAB-capped AZPbBr_3_ NCs confirm the presence of the corresponding capping ligand (Figure 3a). DDAB-capped NCs do not sustain more than one washing cycle, after which a free alkyl carboxylic acid (in this case, oleic acid) is still detected in the DDAB-sample (as alkenyl protons at 5.4 ppm). We thus inspected the C_8_C_12_–PEA-capped NCs in greater detail, since they can be purified at least three times without a notable loss of NC dispersibility. Already twice-washed samples lack any oleate-related signal. ^31^P NMR spectra evidence the surface-bound C_8_C_12_–PEA ligands in solution (Figure 3b) and in the solid state (Figure S10b). The twice-washed C_8_C_12_–PEA–AZPbBr_3_ sample shows only a broad signal centered at approximately −1 ppm from bound C_8_C_12_–PEA (Figure 3b, top). No other signals were detected, excluding free or surface-bound TOPO and DOPA. We also analyzed the NCs synthesized with the addition of alkyl phosphonic acids (Figure 3b, middle), whose ^31^P NMR signal is expected at 25–28 ppm (see the octylphosphonic acid spectrum in Figure S9 and ref (50)). Colloids washed only once exhibit a broad surface-bound C_8_C_12_–PEA signal at −1 ppm and three narrow peaks in the range of 40–60 ppm (absent in twice-washed NC samples), originating from the residual TOPO (49 ppm) and DOPA (57 and 43 ppm for its main impurity). These NC cores were then digested by the addition of DMSO-*d*_6_, liberating surface-bound species, leading to a narrow signal from the free C_8_C_12_–PEA ligand (Figure 3b, bottom) and solvent-related shifts from the TOPO/DOPA species but still no signatures of phosphonic acids. We thus conclude that the small quantities of alkylphosphonic acids used in the synthesis are fully removed upon washing and do not bind to the NC surfaces.

The presence and integrity of the AZ cation in AZPbBr_3_ NCs were characterized with ^1^H solid-state NMR spectra (Figure S11a). The bulk material features two main peaks at 4 and 6 ppm. Additional minor species are resolved at 5 and 8 ppm, although the material was phase-pure according to powder XRD (Figure S12). No NMR signal from possible ring-opened alkyl ammonium was detected. The observed species were also found in the AZPbBr_3_ NCs in solution and solid-state ^1^H measurements, with the addition of the alkyl protons from the ligands at 2 ppm (Figure 3a and Figure S10a). ^207^Pb solid-state NMR is highly sensitive to deviations from a cubic crystal structure in lead-halide perovskites. Experiments on bulk AZPbBr_3_ show a single signal centered at ∼485 ppm with a full width at half-maximum (fwhm) of 13.1 kHz, similar to the cubic FAPbBr_3_ signal (Figure S11b).^51^ The ^207^Pb solid-state NMR signal for C_8_C_12_–PEA-capped AZPbBr_3_ NCs fits very well with the bulk reference, exhibiting a single signal at ∼505 ppm with a fwhm of 16.5 kHz (Figure 3c). Broader peaks in NCs, compared to bulk, have previously been also reported for CsPbBr_3_, likely caused by the increased disorder and higher ion mobility.^51^

### Raman Spectroscopy

Raman spectra of AZPbBr_3_ NCs and AZPbBr_3_ bulk powders confirmed the presence of the AZ cation within the Pb–Br perovskite cage. Beyond the dense and almost featureless spectrum of bands below 150 cm^–1^, characteristic for the Pb–Br framework in 3D lead-bromide perovskites,^52^ both NCs and bulk exhibit a band at ∼308 cm^–1^, a doublet at 807 and 871 cm^–1^, and a band at 1227 cm^–1^, previously assigned to the AZ-cage mode, ring deformation, and ring stretching, respectively.^15^ Only a limited amount of degradation products related to AZ ring opening was detected (Figure S13 and Table S9). Note that some bands remain challenging to assign due to the lack of Raman measurements conducted explicitly on the AZ cation, which is unstable outside the perovskite framework. Notwithstanding these uncertainties, Raman spectroscopy also evidences the AZ cations incorporated in the Pb–Br framework, both in AZPbBr_3_ NCs and bulk.

### Single-QD Spectroscopy

**Figure 4:**
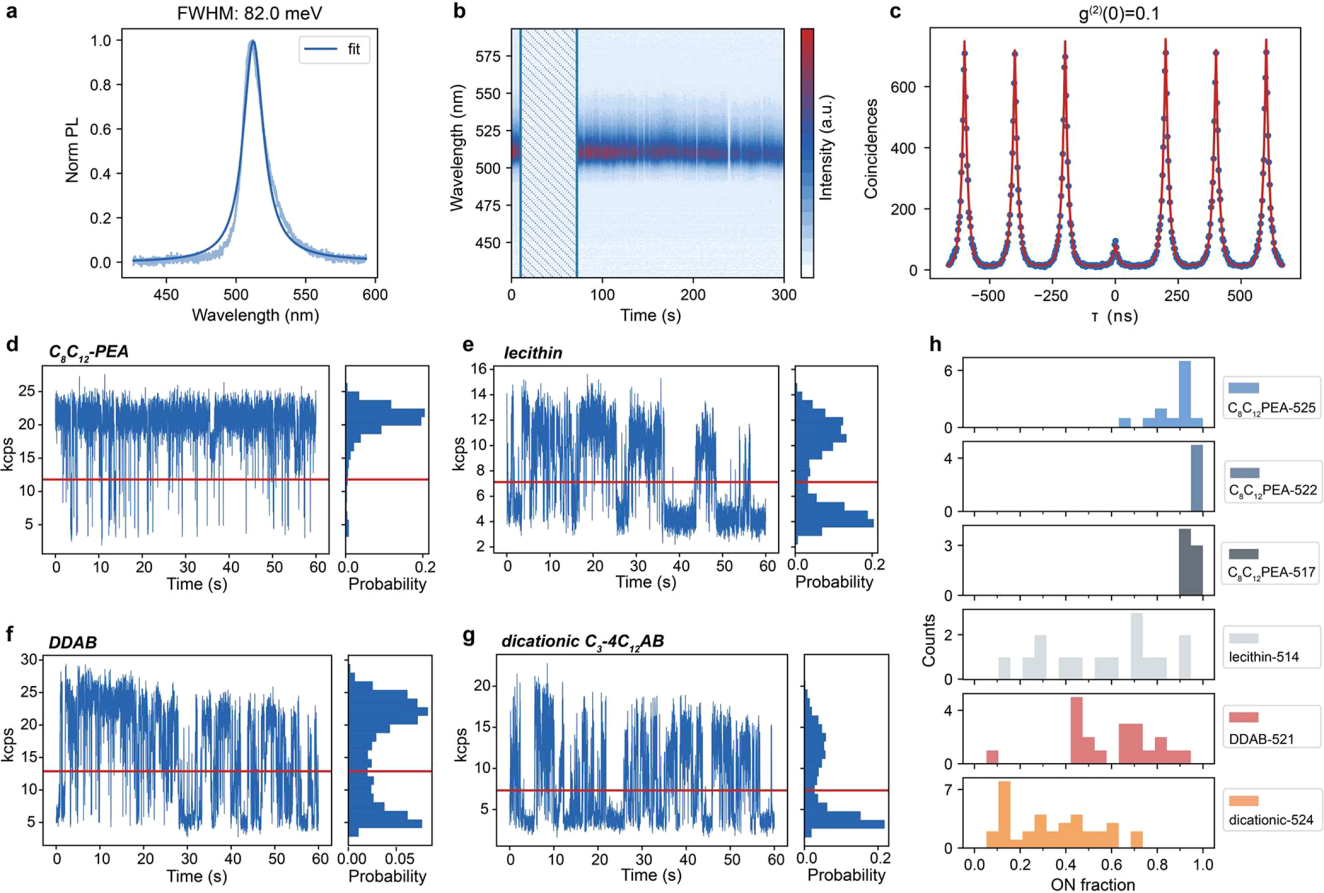
Room-temperature PL of single AZPbBr_3_ QDs with various surface capping ligands. (a) PL spectrum of a single C_8_C_12_–PEA-capped AZPbBr_3_ QD displaying narrow-band emission with a fwhm of 82 meV. (b) Spectra series of a C_8_C_12_–PEA-capped QD exhibiting spectrally stable PL and small intensity variations. The highlighted time period (shaded area without PL spectral detection) corresponds to the acquisition of the second-order correlation function (*g*^(2)^(*t*), shown in panel (c)) and blinking trace by a Hanbury–Brown and Twiss experiment. (c) Second-order photon–photon correlation of a C_8_C_12_–PEA-capped AZPbBr_3_ QD displaying high single-photon purity (*g*^(2)^(0) = 0.1). (d–g) Representative PL blinking traces (10 ms binning time) of a QD capped with branched C_8_C_12_–PEA ligands (panel (d)), lecithin (panel (e)), DDAB (panel (f)), and dicationic amine C_3_–4C_12_AB (panel (g)). (h) Histograms of the fraction of time that single QDs spend in their bright (ON) state. The numbers after the ligand name indicate the ensemble PL central wavelength of the respective sample.

Room-temperature optical properties of AZPbBr_3_ NCs, also referenced here as QDs, due to their quantum-light emission capabilities (vide infra), were examined via single-particle PL spectroscopy in a home-built inverted oil-immersion microscope (see details in the Supporting Information). Such PL studies at the single-QD level unveil basic structure–property relationships,^53^ as well as sample heterogeneities^54^ and temporal fluctuations^55,56^ of emitters, which are otherwise unresolved in the ensemble spectra. Figure 4a shows a single-particle PL spectrum of an AZPbBr_3_ QD capped by C_8_C_12_–PEA ligands. Fitting a Lorentz function to the experimental data returns an emission peak centered at 512 nm and an fwhm of 82 meV, demonstrating the spectrally narrow emission of the individual AZPbBr_3_ QDs. Measurements were performed in a nitrogen atmosphere for extended spectral photostability, as evidenced in the PL spectra series in Figure 4b. AZPbBr_3_ QDs exhibited high single-photon purity, as confirmed by a strongly suppressed peak at a zero delay time in the second-order photon–photon correlation function *g*^(2)^(*t*) (Figure 4c). The high single-photon purity with g^(2)^(0) = 0.1 is on par with FAPbBr_3_ and MAPbBr_3_ QDs capped by the same C_8_C_12_–PEA ligand.^25^

Furthermore, we quantify the PL intensity fluctuations, termed “PL blinking”, i.e., the stochastic switching between a bright (ON) and a dimmed (OFF) state. PL blinking roots in a photoinduced charge trapping at surface defects, possibly mediated by efficient Auger–Meitner recombination, and could be strongly affected by the QD surface passivation.^57^ The fraction of time spent in the ON state (ON fraction) is therefore a suitable metric of the surface quality for the nanomaterial under study at the single-QD level. Recently, we demonstrated that the C_8_C_12_–PEA ligands stabilize hybrid organic–inorganic lead-halide perovskite QDs and enable emission at the single-particle level with >90% ON fraction.^25^ A blinking trace from a single C_8_C_12_–PEA-capped AZPbBr_3_ QD (Figure 4d) also exhibits a high ON fraction (∼95%), representative of the high ON fraction (typically >85%) for AZPbBr_3_ QDs with this ligand capping (Figure 4h). We then surveyed the blinking behavior of QDs capped by various alternative and post-synthetically attached ligands, i.e., zwitterion lecithin (Figures 4e), monocationic DDAB (Figure 4f), and dicationic C_3_–4C_12_AB (Figure 4g). Although C_8_C_12_–PEA and lecithin are both zwitterionic phospholipids, lecithin-capped QDs exhibit longer OFF events and a significantly smaller ON fraction than those of C_8_C_12_–PEA-capped QDs, symptomatic for the compromised surface passivation of the former. We attribute the superior performance of C_8_C_12_–PEA to the better fit of its primary ammonium binding group into the A-cation site at the QD surface.^25,30^ Likewise, blinking traces of QDs capped by one of the two quaternary ammonium ligands, DDAB or C_3_–4C_12_–AB, displayed ON fractions smaller than those of the C_8_C_12_–PEA-capped QDs. We further observed that all samples, except C_8_C_12_–PEA-capped QDs, feature a large QD-to-QD variation in the ON fraction (Figure 4h). This could be assigned to the sample preparation procedure needed for single-QD spectroscopy, which requires a strong dilution (by a factor of ∼50 000) of the colloids.

The dilution step can also alter the QD morphology^58^ and induce a blue shift of the PL energy (Figure S14). Occurring under water-free and inert conditions, structural and optical alterations upon dilution are rationalized considering ligand desorption enhanced by the dynamic ligand binding observed in ionic perovskite QDs.^59^ Dilution-induced alterations can also explain the deviation of ON fractions for the different ligands, despite comparably high PL QYs in the undiluted ensemble (Figure S14). Single-particle spectroscopy thus acts as a stress test for ionic QDs with inherently dynamic ligand binding that is better endured by C_8_C_12_–PEA-capping. The reduced blinking for such a ligand formulation could be exploited for the realization of single-photon sources without the loss in the single-photon purity.^60^

**Figure 5:**
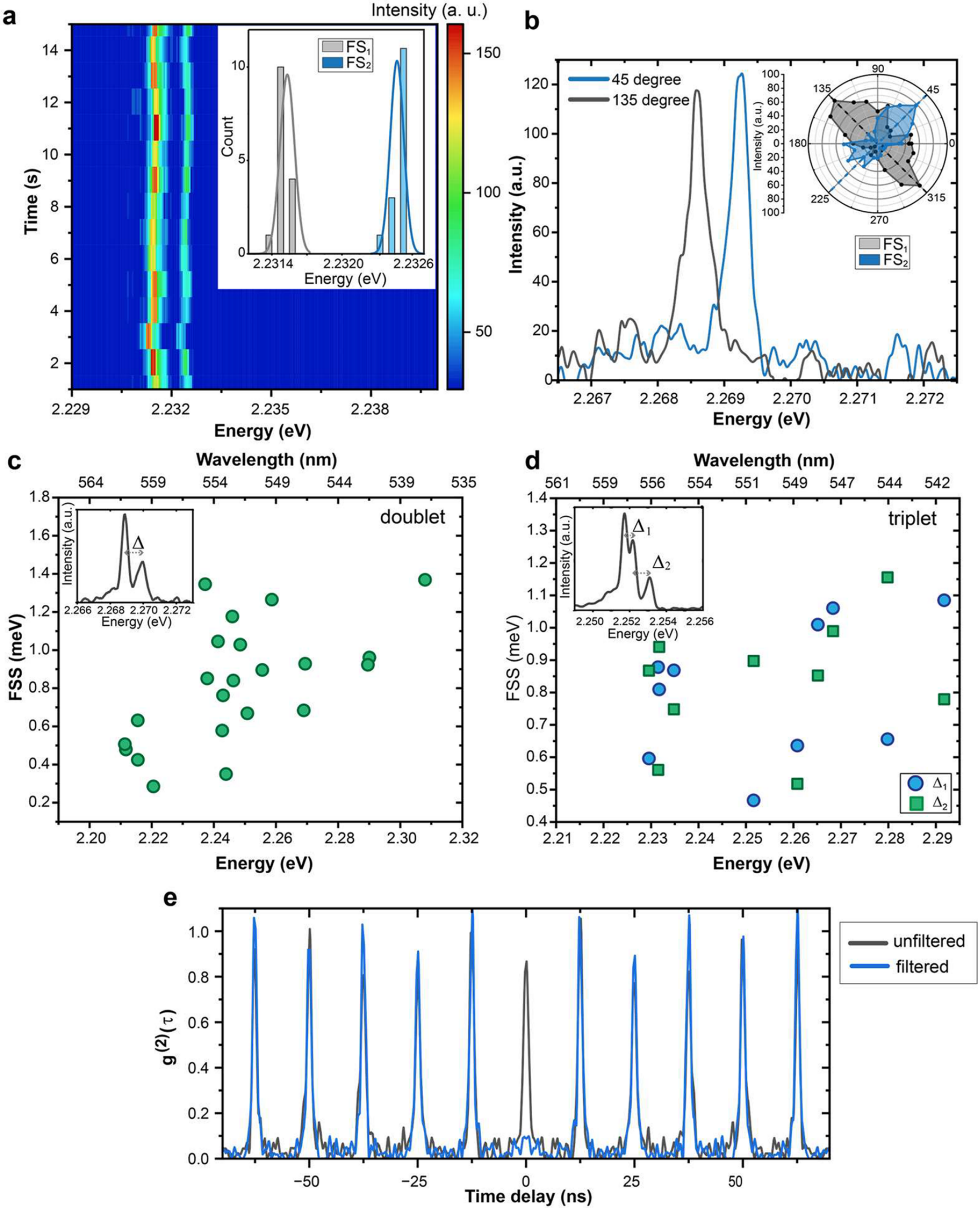
Exciton fine-structure of single AZPbBr_3_ QDs at 4 K. (a) Time series of a single QD (1 s integration time and 1800 g/mm grating); sub-meV spectral diffusion allows to resolve the exciton fine structure. This QD exhibits a doublet fine structure with peaks denoted as FS_1_ and FS_2_. The inset shows an associated histogram of the peak energies (bars), fitted with a Gaussian function (lines). (b) The PL spectra of one single QD measured at two different angles of a linear polarizer in the detection path: 45° (blue) and 135° (gray); this QD has a doublet exciton fine structure. The inset shows a polar plot with the respective PL intensities, as a function of the polarizer angle; both doublet sublevels exhibit highly linear polarization, oriented perpendicular to each other; (c, d) Fine-structure splitting energy (FSS) for all of the single QDs (capped with DDAB or C_8_C_12_–PEA ligands) exhibit doublet sublevels (panel (c)) or triplet sublevels (panel (d)). The insets in panels (c) and (d) show representative spectra for doublet and triplet exciton fine structures, respectively. (e) Second-order correlation function *g*^(2)^(τ) of a single AZPbBr_3_ QD under 0.3 μJ/cm^2^ before (dark gray line) and after (blue line) spectrally filtering out the biexciton emission by a tunable short-pass filter.

While room-temperature single-QD experiments unveiled the optimal ligand choice, the intrinsic electronic properties of the semiconductor core are probed at cryogenic temperatures. At 4 K, the perturbation by phonons via exciton–phonon coupling is highly suppressed, enabling observation of the exciton fine structure arising from electron–hole exchange interaction as well as emission from exciton complexes. We studied single AZPbBr_3_ QDs capped with C_8_C_12_–PEA, DDAB, or lecithin ligands. Single QDs with ensemble QD sizes from ∼7–9 nm exhibit exciton PL bands in the range of 2.31–2.20 eV (537–564 nm), with a fwhm ranging from 0.2 to 0.8 meV (where 0.2 meV is our setup resolution). Among the studied ligand systems, only DDAB- and C_8_C_12_–PEA-capped single QDs exhibit a spectrally stable multiline exciton spectrum with sub-meV spectral diffusion (Figure 5a). The multiline spectrum is ascribed to the bright triplet character of excitonic emission in lead-halide perovskites.^7,61^ By introducing a linear polarizer in the collection path and recording the angle-dependent exciton intensities, we obtained the polarization profile typical for a bright triplet exciton: individual exciton sublevels are highly linearly polarized and orthogonal to each other, as in the example displayed in Figure 5b. Across various single QDs, we observed both doublet and triplet exciton fine structures (see insets of Figures 5c and 5d). Splitting energies of doublet (Δ) and triplet (Δ_1_ and Δ_2_) exciton fine structures are plotted as a function of the exciton energy in Figures 5c and 5d, respectively. Splitting energies vary from 0.2 to 1.4 meV for QDs with PL peak ranging from 2.21 to 2.31 eV. In addition to a systematic trend of increasing splitting energies with increasing exciton energy, large variations in splitting energy are observed for a given exciton energy. This variation was also reported in other lead halide perovskite QDs,^6,7,61−65^ and suggests that the exciton fine-structure splitting is sensitive to the shape anisotropy of the studied QDs. In general, exciton properties of AZPbBr_3_ QDs are comparable to CsPbBr_3_^7,66,67^ and FAPbBr_3_ QDs.^61,68^ Compared to the C_8_C_12_–PEA- or DDAB-capped single QDs, lecithin-capped QDs exhibit stronger spectral diffusion (∼10 meV, see Figure S15), consistent with their more-pronounced PL blinking and lower ON fraction at room temperature.

To quantify the quantum correlations among photogenerated exciton complexes, we drive the single QDs under high excitation fluence, hereby obtaining emission also from trion (X*) and biexciton (XX); see one example in Figure S16a. As the excitation fluence increases from 0.003 μJ/cm^2^ to 0.09 μJ/cm^2^, two additional peaks emerge on the lower-energy side of the exciton emission spectrum, which are assigned to the trion (red-shifted from *E*_*x*_ by 25 meV) and biexciton (red-shifted from *E*_*x*_ by 40 meV). Summarizing the results from all studied single QDs, binding energies of trion (Δ_X*_ = *E*_*X*_ – *E*_X*_) and biexciton (Δ_*XX*_ = *E*_*X*_–*E*_*XX*_) are plotted as a function of the exciton energies (see Figure S16b). Δ_X*_ increases from 10 to 25 meV for exciton energies increasing from 2.20 to 2.30 eV, and Δ_*XX*_ increases from 30 to 40 meV for exciton energies increasing from 2.24 to 2.30 eV. Qualitatively, the observed trend of increasing Δ_X*_ or Δ_*XX*_ with increasing exciton energy is universal in semiconductor QDs^69,70^ as the Coulomb interaction among photogenerated charge carriers is steadily enhanced with decreasing QD size. Quantitatively, the size-dependent trends of Δ_X*_ and Δ_*XX*_ are similar to those reported for FAPbBr_3_ QDs.^71^ Utilizing the obtained knowledge of Δ_*XX*_ in AZPbBr_3_ QDs, we performed single-QD antibunching experiments under high excitation fluence (0.3 μJ/cm^2^) with and without filtering the biexciton emission. When the spectrum is unfiltered (Figure 5e, dark gray line), no antibunched emission was observed, i.e., *g*^(2)^(0) ≈ 1, suggesting very efficient biexciton emission at 4 K. When the biexciton emission is discarded by spectral filtering (Figure 5e, blue line), emission is characterized by a regular stream of single photons (*g*^(2)^(0) ≈ 0, within the noise floor of ∼0.1). In addition, all the studied individual AZPbBr_3_ QDs feature a monoexponential PL decay with lifetimes between 400 and 1600 ps, as shown in Figure S17. These lifetimes are much longer than reported in CsPbBr_3_ QDs, suggesting the absence of a pronounced giant oscillator strength,^7^ probably related to the softer and more dynamic lattice of the AZPbBr_3_ QDs. The room-temperature PL decay curves (Figure S18) reveal a reduction in the radiative rates with decreasing NC size. For CsPbBr_3_ NCs, such dependence was previously explained by the size-dependent thermal mixing with optically forbidden excitonic transitions.^72^

## Conclusions

In summary, we have presented a room-temperature colloidal synthesis of monodisperse and quantum-confined AZPbBr_3_ NCs with bright emission, size-tunable PL peaks from 498 to 530 nm, and a locally distorted cubic crystal. The stabilization of the AZ cation is confirmed by NMR and Raman spectroscopy. At the single-particle level, AZPbBr_3_ QDs capped with DDAB or C_8_C_12_–PEA are as robust as analogously synthesized MAPbBr_3_ and FAPbBr_3_ QDs reaching high single-photon purity and suppressed blinking behavior at room temperature, and exhibiting bright triplet exciton character of the single-exciton emission at 4 K. This report on the synthesis of colloidal AZPbBr_3_ NCs can inspire follow-up investigations of such NCs, for example, further exploring potentials for compositional engineering (mixed cations or anions) and integration into devices such as color enhancers or blue-to-green down-converters.

## Methods

### Safety Statement

No unexpected or unusually high safety hazards were encountered.

### Synthesis of AZPbBr_3_ NCs

#### Stock Solutions

*Aziridine stock solution (0.15 M)* was prepared as follows: 15.5 μL of aziridine was dissolved in 2 mL of anhydrous chloroform and stored in a refrigerator. For *PbBr_2_-TOPO stock solution (0.04 M)*, PbBr_2_ (1 mmol, 376 mg) and TOPO (5 mmol, 2.15 g) were dissolved in octane (5 mL) at 100 °C, followed by dilution with hexane (20 mL) and filtering through a 0.2 μL PTFE filter before use. *DOPA stock solution (0.57 M)* was prepared by dissolving 0.8 mL of DOPA in 3.2 mL hexane. *OA stock solution (0.515 M)* was prepared by dissolving 0.8 mL of OAc in 3.6 mL of hexane. *EtHAc stock solution (0.31 M)* was prepared by dissolving 0.2 mL of EtHAc in 3.8 mL of hexane. *Erucic acid stock solution (0.56 M)* was prepared by dissolving 383 mg of EAc in 2 mL hexane. *Alkylphosphonic acid stock solution (alkylPAc, 0.257 M)* was prepared by dissolving alkyl phosphonic acid in anhydrous toluene (hexyl-, octyl-, decyl-, or dodecylphosphonic acid). C_10_PAc and C_12_PAc dissolve in toluene only upon heating to 80 °C, and the stock solution should be preheated before use. *DDAB stock solution (100 mg/mL*, *0.215 M)* was prepared by dissolving 300 mg of DDAB in 3 mL of anhydrous toluene. *C*_8_*C_12_-PEA stock solution (50 mg/mL)* was prepared by dissolving 100 mg of C_8_C_12_–PEA in 2 mL of distilled mesitylene. *Lecithin stock solution (50 mg/mL)* was prepared by dissolving 200 mg of lecithin in 4 mL hexane. *C_3_-4C_12_AB stock solution (100 mg/mL)* was prepared by dissolving 100 mg C_3_-4C_12_AB in 1 mL of mesitylene.

#### Synthesis

In a 25 mL one-neck flask, PbBr_2_-TOPO stock solution was combined with additional hexane. Then, a desired volume of DOPA, alkyl-PAc, or OAc (or other carboxylic acids) stock solution was added. Under vigorous stirring (1100 rpm), aziridine in chloroform was swiftly injected into the reaction mixture. After 20 s to 3 min, a stock solution of ligands (DDAB or C_8_C_12_–PEA, lecithin, or C_3_-4C_12_AB) is added to initiate the ligand exchange on the NC surface. Within 2–4 min after the addition of ligands, the crude solution was concentrated by evaporating hexane on a rotary evaporator down to <0.5 mL of residual solvent. The NCs were precipitated from the concentrated colloid by adding nonsolvent. Specific volumes of stock solutions are indicated in Table S1 for all reactions presented in this work.

#### Purification

For *DDAB ligands*, NCs were purified using acetone (crude solution:nonsolvent 1:2 (v/v)), followed by solubilization of the obtained NCs in cyclohexane. For *C_8_C_12_-PEA ligands*, ethyl acetate and acetonitrile mixture (1:1) were used (crude solution:nonsolvent, 1:1 (v/v)), followed by solubilization of the obtained NCs in cyclohexane. For lecithin ligands*,* ethyl acetate and acetonitrile mixture (1:1) was used (crude solution: nonsolvent, 1:1 (v/v)), followed by solubilization of the obtained NCs in anhydrous toluene. For dicationic *C_3_-4C_12_AB ligands*, acetone was used as a nonsolvent. In the case of introducing *phosphonic acids* in the synthesis, first, the crude solution was centrifuged, then a white precipitate was discarded, and the obtained supernatant was concentrated by evaporating on a rotary evaporator down to <0.5 mL of residual solvent, followed by purification with an ethyl acetate/acetone mixture (1:1), and the obtained NCs were dissolved in toluene. Both DDAB and C_8_C_12_–PEA-capped AZPbBr_3_ NCs are stable for several months in darkness under ambient conditions. The described synthesis is sensitive to the value of room temperature. Deviation in room temperature (in our case, 21 °C) by 2–3 °C leads to the shift of the PL maximum by 2–4 nm.

### Ex-Situ Absorption and PL Spectroscopy

UV-vis absorption spectra were collected using a Jasco Model V770 spectrometer operated in transmission mode. A spectrofluorimeter (Horiba Jobin Yvon, Model Fluoromax 4) that was equipped with a PMT detector was used to acquire steady-state PL spectra from solutions. The excitation wavelength was 400 nm, provided by a 150 W xenon lamp dispersed with a monochromator. Measured intensities were corrected to consider the detector’s spectral response. The QY of the solutions was measured with a quantum yield spectrometer (Hamamatsu, Model Quantaurus-QY Absolute PL) that was equipped with an integrating sphere. Time-resolved PL traces were acquired in solution by using a FluoTime300 spectrometer from PicoQuant. Samples were excited using a 355 nm detector; the detection was set at the PL peak maximum for each sample.

### In-Situ Absorption Setup

The reactions were carried out in a modified commercial cuvette holder (CVH100; Thorlabs). The absorption spectra were recorded in transmission mode with a deuterium-tungsten light source (Ocean Optics, Model DH-2000-BAL-TTL-24 V) and a broad-band spectrometer (OceanInsight, Model HDX-XR). The time resolution in these measurements was set to 50 ms. Custom-developed batch analysis scripts (written in Python) were employed for the data analysis and fitting.

### Electron Microscopy Characterization

Transmission electron microscopy (TEM) and scanning transmission electron microscopy (STEM) images were collected by using a JEOL Model JEM-2200FS microscope operated at 200 kV. HR HAADF-STEM was carried out using a probe-aberration-corrected FEI Titan Themis system that was operated at 300 kV, using a beam current of ∼1 pA. High-resolution images were obtained by summing up 3–5 frames.

### SAXS Measurements

SAXS experiments were carried out on a benchtop Bruker Nanostar (Bruker AXS GmbH, Karlsruhe, Germany) using the K_α_-line of a microfocused X-ray Cu source with a wavelength of 1.5406 Å. The beam was collimated using a 0.3 mm pinhole, leading to a beam diameter of ∼0.4 mm at the sample position. The sample–detector distance was set to 107 cm and further calibrated with silver behenate, achieving a resolvable *q*-range of 0.07 nm^–1^ ≤ *q* ≤ 2.3 nm^–1^. For selected samples, an additional measurement was performed at a sample-to-detector distance of 27 cm, further calibrated with silver behenate, and combined with previous measurements to obtain an extended q-range of 0.07 nm^–1^ ≤ *q* ≤ 10 nm^–1^. The length of the scattering vector *q⃗* is defined as *q* = 4π sin 2θ/λ, where 2θ as the scattering angle and λ the wavelength of the X-ray source; the scattered intensity was recorded on a gaseous avalanche-based detector (VÅNTEC-2000, Bruker AXS) with 2048 × 2048 pixels and a pixel size of 68 μm × 68 μm. The scattering patterns were recorded at room temperature under moderate vacuum conditions (10^–2^ mbar) to limit air scattering.

Calibration of the scattering vector length *q* and estimation of the instrumental resolution Δ*q* = 0.25 nm^–1^ were done by measuring the first diffraction peak of a silver behenate sample and calculating its width. Each sample was sealed in a quartz capillary and mounted in the sample chamber. The scattering intensity was recorded for 700 s for each sample. The intensity of the semitransparent beamstop from empty beam scans was used for transmission calibration. The scattered intensity was extracted, azimuthally averaged, and integrated over each *q*-value using the Bruker software DIFFRAC.EVA (Bruker AXS, version 4.1). The 1D data was transmission-corrected and background-subtracted from the scattering of the respective solvent, polymers, and the empty quartz capillary using an in-house data pipeline operating under Matlab 2022.

The final scattering pattern was then fitted using the parallelepiped model. For more details, we refer to the section “Small-Angle X-ray Scattering (SAXS) Measurements” in the Supporting Information.

### Synchrotron WAXTS Measurements

X-ray total scattering measurements on AZPbBr_3_ NCs were performed at the X04SA-MS beamline of the Swiss Light Source (Paul Scherrer Institute, Villigen, Switzerland)^73^ and at the Swiss–Norwegian beamline (BM01) of the European Synchrotron Radiation Facility (ESRF, Grenoble, France). For more details, we refer to the section “Synchrotron X-ray Total Scattering Measurements” in the Supporting Information.

### NMR Measurements

#### Solid-State Magic Angle Spinning Nuclear Magnetic Resonance (MAS NMR)

NMR experiments were performed under ambient conditions on a 16.4T Bruker Avance IIIHD spectrometer (Bruker Biospin, Fällanden, Switzerland), that was equipped with a 2.5-mm trippel-channel solid-state probe head. All samples were ground into a fine powder and densely packed into ZrO_2_ rotors. The spinning frequency was set to 20 kHz. ^1^H, ^31^P, and ^207^Pb NMR chemical shifts were externally referenced to TMS, 85% H_3_PO_4_ in H_2_O, and PbMe_4_. The ^1^H excitation pulse was set to 2.5 μs. The ^31^P excitation pulse was set to 5.6 μs, and proton decoupling with a Spinal64 sequence was performed during the detection. For ^207^Pb, a Hahn echo pulse-sequence was used for all measurements with an echo delay of 0.9 ms. The radio-frequency (rf) field of the echo pulses was set to 19.8 kHz.

#### Solution NMR

Solution ^1^H and ^31^P NMR spectra were recorded on a 11.7 T Bruker Avance IIIHD spectrometer (Bruker Biospin, Fällanden, Switzerland). The instrument was equipped with a BBO cryogenic probe. The sample temperature was set to 298 K. ^1^H spectra were acquired using a one-pulse sequence with a 4 μs excitation pulse and a 1 s recycle delay. ^31^P spectra were acquired using a one-pulse excitation pulse (14 μs) and proton decoupling with a 2 s recycle delay. All spectra were referenced externally to TMS (^1^H) and 85% H_3_PO_4_ in H_2_O (^31^P).

### Powder X-ray Diffraction

Powder X-ray diffraction (XRD) patterns were collected in transmission mode with a STADI P diffractometer (STOE&Cie GmbH) that was equipped with a curved Ge (111) monochromator (Cu Kα_1_ = 1.54056 Å) and a silicon strip MYTHEN 1K Detector (Fa. DECTRIS). For the measurement, the ground powder was placed between the adhesive tape.

### Raman Spectroscopy

Raman spectra were acquired using a Horiba LabRAM HR Evolution confocal microscope. An objective lens (100× magnification, 0.9 NA) was used to induce and collect Raman-scattered light from micrometer-scale regions of the samples, using laser excitation at 785 nm (continuous wave, 30 mW). No sample degradation was observed during or after the typical acquisition time of ∼25 s. For more details, we refer to the section “Raman Spectroscopy” in the Supporting Information.

### Single-QD Spectroscopy

#### Sample Preparation (Room Temperature)

The following steps were performed in a glovebox that is kept under a nitrogen atmosphere and employing dry and filtered octane (Acros Organics, 99+% extra dry), cyclohexane (Acros Organics, 99.5% extra dry), and toluene (Acros Organics, 99.85% extra dry over molecular sieve). AzPbBr_3_ NCs were diluted by a factor of 1500–40 000, depending on initial concentration and solvent. Samples were diluted in octane (in the case of C_3_–4C_12_AB-capped NCs), cyclohexane (in the case of DDAB-capped NCs), or toluene (C_8_C_12_PEA or lecithin-capped NCs). Subsequently, 100 μL of this solution was spin-coated onto a cover glass (Thorlabs, 170 ± 5 μm thickness and 25 mm diameter) at 150 rps for one min or 50 rps for 80 s. The samples were then placed in a home-built sample holder filled with nitrogen to preclude moisture-induced (water and/or oxygen) degradation during the measurements for room temperature experiments.

#### Room-Temperature Measurements

Room-temperature single-particle fluorescence measurements were performed on a home-built uPL inverted microscope. The setup consists of a 405-nm pulsed laser (PicoQuant, 10 MHz repetition rate, <50 ps pulse width, <100 W/cm^2^) focused (1/e^2^ = 1 μm) using an oil immersion objective (1.3 NA) onto the sample mounted on a XYZ-stage (SmarAct). The emitted light is collected by the same objective and passed through a dichroic mirror and a long-pass filter (both 450-nm cut-on wavelength). To record the spectrum, the emitted light is sent to a monochromator coupled to an EMCCD (Princeton Instruments, one frame per second). PL intensity time traces and second-order photon–photon correlations are obtained by sending the emitted light to a Hanbury-Brown and Twiss (HBT) setup consisting of a 50/50 beam splitter, two APDs (Excelitas, 250 ps time resolution), and a TCSPC module (PicoQuant, HydraHarp).

#### Cryogenic Measurements (at 4 K)

For single-QD spectroscopy at 4 K, a home-built micro-PL setup is used. The samples were mounted on XYZ nanopositioning stages inside an evacuated liquid-helium closed-loop cryostat (Montana Instruments) and cooled to a targeted temperature of 4 K. Single NCs were excited using a fiber-coupled excitation laser at an energy of 2.585 eV (480 nm) with a repetition rate of 80 MHz (Toptica, <200 fs pulses), which is focused (1/e^2^ diameter = 2.4 μm) on the sample by a microscope objective (0.8 NA, 100× magnification). Typical energy densities used to excite single NCs were 0.003–0.008 μJ/cm^2^. The emitted light is collected by the same objective and passed through a dichroic mirror (long-pass, cut-on wavelength of 488 nm) and a long-pass filter at 500 nm. A monochromator (Princeton Instruments, 0.75 m) coupled to a back-illuminated CCD camera is used for spectra measurements. Spectra are measured with a grating featuring 1800 (or 300) lines/mm, blaze 500 nm, resulting in a 0.2 (or 1) meV spectral resolution.

### Computational Methods

For details of the computational study, we refer to the section “Computational Methodology” in the Supporting Information.

## Supporting Information Available

Additional details on materials and methods including complete description of the synthetic procedure, TEM and STEM images, Raman spectroscopy measurements, NMR, additional SAXS, WAXTS and single-dot spectroscopy data, and the computational study (PDF)
