# Supplementary material for: Colloidal Aziridinium Lead Bromide Quantum Dots
Source: ACS Nano. 2024 Feb 6;18(7):5684–97. doi: 10.1021/acsnano.3c11579 (PMC10883123; doi:10.1021/acsnano.3c11579)
Supplement: Supplementary file 1 — nn3c11579_si_001.pdf [file nn3c11579_si_001.pdf]

Supporting Information

Colloidal aziridinium lead bromide quantum dots

Maryna I. Bodnarchuk, [Δ,#,*] *Leon G. Feld,* [#,Δ] *Chenglian Zhu,* [#,Δ] *Simon C. Boehme,* [#,Δ] *Federica Bertolotti,* [⸸] *Jonathan Avaro,* [◊] *Marcel Aebli,* [#,Δ] *Showkat Hassan Mir,* [⌂] *Norberto Masciocchi,* [⸸] *Rolf Erni,* [§] *Sudip Chakraborty,* [⌂] *Antonietta Guagliardi,* [‡] *Gabriele Rainò,* [#,Δ] *Maksym V. Kovalenko* [#,Δ,♦,*]

[Δ] Laboratory for Thin Films and Photovoltaics, Empa, Swiss Federal Laboratories for Materials Science and Technology, Dübendorf 8600, Switzerland

[#] Institute of Inorganic Chemistry, Department of Chemistry and Applied Biosciences, ETH Zürich, Zürich 8093, Switzerland

[♦] SKKU Institute of Energy Science and Technology (SIEST), Sungkyunkwan University, Suwon 16419, South Korea

[§] Electron Microscopy Center, Empa, Swiss Federal Laboratories for Materials Science and Technology, Dübendorf 8600, Switzerland

[◊] Centre for X-ray Analytics & Laboratory for Biomimetic Membranes and Textiles, Empa, Swiss Federal Laboratories for Materials Science and Technology, St. Gallen 9014, Switzerland

[⸸] Department of Science and High Technology and To.Sca.Lab., University of Insubria, via Valleggio 11, 22100 Como 22100, Italy

[‡] Istituto di Cristallografia and To.Sca.Lab, Consiglio Nazionale delle Ricerche, via Valleggio 11, Como 22100, Italy

[⌂] Materials Theory for Energy Scavenging (MATES) Lab, Harish-Chandra Research Institute (HRI)Allahabad, ***A*** C.I. of Homi Bhabha National Institute (HBNI), Chhatnag Road, Chhatnag Road, Jhunsi, Prayagraj (Allahabad) 211019, India

*maryna.bodnarchuk@empa.ch, mvkovalenko@ethz.ch

Chemicals

Lead bromide ($PbBr_2$, 99.999%), diisooctylphosphinic acid (DOPA, 90%), oleic acid (OA, 90%), 2-Ethylhexanoic acid (EtHAc), erucic acid (EAc), hexylphosphonic acid (C_6PAc, 95%), octylphosphonic acid (C_8PAc, 97%), decylphosphonic acid ($C_{10}PAc$, 97%), dodecylphosphonic acid ($C_{12}PAc$), didodecyldimethylammonium bromide (DDAB), acetone, ethyl acetate, toluene, cyclohexane, and acetonitrile were purchased from Sigma Aldrich. Lecithin (>97% from soy) and n-octane were purchased from Carl Roth. Trioctylphosphine oxide (TOPO, min. 90%) was purchased from Strem Chemicals. Aziridine (95 %) was purchased from Ochem Incorporation. Mesitylene (99 %, ME) was purchased from Acros. All chemicals were used as received.

2-octyldodecylphosphoethanolamine (C_8C_{12}-PEA) has been synthesized according to Ref.[1]

Propanediyl-1,3-N, N-bis (didodecylmethylammonium bromide) ligand was synthesized according to Ref.[2] and is further depicted as C_3-$4C_{12}$AB.

Synthesis of $AZPbBr_3$ NCs

Table S1. *Reaction conditions of the synthesis of $AZPbBr_3$ NCs reported in the Main Text and Supporting Information.*

Figures	$PbBr_2$-TOPO stock solution (ml)	solvent (ml)	DOPA stock solution (ml)	acid stock solution (ml)	additional alkyl PAc stock solution (ml)	AZ stock solution, (µl)	growth time, (s)	Ligand	ligand stock solution (µl)	Final PL (nm)
standard	0.65	6	0.1	0.15 EtHAc	-	44	30	DDAB	30	521
Fig. 1	0.65	3	0.1	0.2 OAc	0.175 C_{10}PAc	44	20	C_8C_{12}-PEA	100	499
Fig. 1	0.65	3	0.1	0.2 OAc	0.2 C_{10}PAc	44	20	C_8C_{12}-PEA	50	502
Fig. 1, S2d	0.65	3	0.1	0.2 OAc	0.175 C_{10}PAc	44	20	DDAB	30	504
Fig. 1, S2d	0.65	3	0.1	0.2 OAc	0.15 C_{10}PAc	44	20	DDAB	30	507
Fig. 1	0.65	3	0.1	0.2 OAc	0.175 C_{10}PAc	44	140	C_8C_{12}-PEA	60	509
Fig. 1	0.65	3	0.1	0.2 OAc	0.175 C_{10}PAc	44	90	DDAB	40	513.5
Fig. 1	0.65	3	0.1	0.2 OAc	0.175 C_{10}PAc	44	140	DDAB	30	515
Fig. 1, S2a	0.65	3	0.1	0.2 OAc	0.175 C_8PAc	44	90	DDAB	30	518
Fig. 1	0.65	3*	0.1	0.2 OAc	0.15 C_8PAc	44	80	lecithin	0.3	520.5
Fig. 1, S2b	0.65	3	0.1	0.2 EAc	-	44	20	DDAB	30	523
Fig. 1	0.65	3	0.1	0.2 OAc	-	44	30	C_8C_{12}-PEA	15	525
Fig. 1, S1	0.65	3	0.1	0.2 EAc	-	44	180	DDAB	30	528
Fig. 1	0.65	1.5	0.1	0.2 EAc	-	44	475	DDAB	30	530
Fig. S2a	0.65	3	0.1	0.2 OAc	0.175 C_8PAc	44	20	DDAB	40	511
Fig. S2a	0.65	3	0.1	0.2 OAc	0.175 C_8PAc	44	30	DDAB	10	516
Fig. S2a	0.65	3	0.1	0.2 OAc	0.175 C_8PAc	44	105	DDAB	10	519
Fig. S2a	0.65	3	0.1	0.2	0.175 C_8PAc	44	240	DDAB	10	521
Fig. S2b, S2d, S3	0.65	3	0.1	0.36 EtHAc	-	44	30	DDAB	30	521
Fig. S2b, S5	0.65	3	0.1	0.2 OAc	-	44	20	DDAB	30	521
Fig. S2c	0.65	1.5	0.1	0.36 EtHAc	-	44	20	DDAB	30	522
Fig. S2c	0.65	3	0.1	0.36 EtHAc	-	44	20	DDAB	30	522
Fig. S2c	0.65	6	0.1	0.36 EtHAc	-	44	20	DDAB	30	521
Fig. S2c	0.65	15	0.1	0.36 EtHAc	-	44	20	DDAB	30	519
Fig. S2d	0.65	3	0.1	0.2 OAc	0.1 C_{10}PAc	44	20	DDAB	30	509
Fig. S1, S5, Tab. S2	0.65	3	0.1	0.2 OAc	0.15 C_{12}PAc	44	10	C_8C_{12}-PEA	25	501

Fig. S1, S5, Tab. S2	0.65	3	0.1	0.2 OAc	0.15 C_{12}PAc	44	10	C_8C_{12}-PEA	18	505
Fig. S1, S5, Tab. S2	0.65	3	0.1	0.2 OAc	0.15 C_{10}PAc	44	10	C_8C_{12}-PEA	15	508
Fig. S1, S5, Tab. S2	0.65	3	0.1	0.2 OAc	0.15 C_{10}PAc	44	60	C_8C_{12}-PEA	15	512
Fig. S1, S5, Tab. S2	0.65	3	0.1	0.2 OAc	0.15 C_{10}PAc	44	90	C_8C_{12}-PEA	15	514
Fig. S1, S5 Tab S2	0.65	3	0.1	0.2 OAc	0.15 C_8PAc	44	25	DDAB	20	516
Fig. S1, S5, Tab. S2	0.65	3	0.1	0.3 EAc	-	44	12	DDAB	30	523
Fig. S5, Tab. S2	0.65	3	0.1	0.2 EAc	-	44	180	DDAB	30	529
Fig. S3	0.65	3	0.1	0.2 OAc	0.15 C_{12}PAc	44	20	C_8C_{12}-PEA◊	27.5	498
Fig. S3	0.65	3	0.1	0.2 OAc	0.2 C_{10}PAc	44	20	C_8C_{12}-PEA◊	25	502
Fig. S3	0.65	3	0.1	0.2 OAc	0.175 C_{12}PAc	44	110	C_8C_{12}-PEA◊	30	505
Fig. S3	0.65	3	0.1	0.2 OAc	0.15 C_{10}PAc	44	20	C_8C_{12}-PEA	15	508
Fig. S3	0.65	3	0.1	0.3 EAc	-	44	20	DDAB	30	513
Fig. S3	0.65	3*	0.1	0.2 OAc	0.15 C_{10}PAc	44	80	C_8C_{12}-PEA	15	517
Fig. S3	0.65	3	0.1	0.2 OAc	-	44	30	C_3-4C_{12}AB	30	525
Fig. S3	0.65	3	0.1	0.2 EAc	-	44	180	DDAB	30	529

* - mesitylene was used as a solvent instead of hexane
C_8C_{12}-PEA◊ - C_8C_{12}-PEA stock solution with a concentration of 100 mg/mL

Synthesis of bulk $AZPbBr_3$

Powdered **bulk $AZPbBr_3$** was synthesized according to the method reported in Ref.[3]

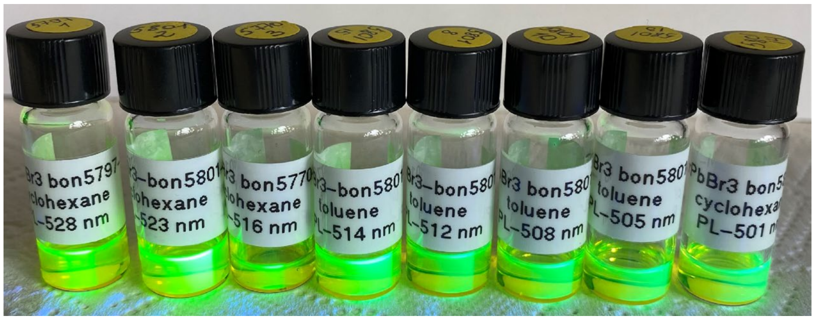

Figure S1. *Photograph of $AZPbBr_3$ NC colloidal solutions with PL peaks ranging from 501 nm to 528 nm (from right to left) under UV light.*

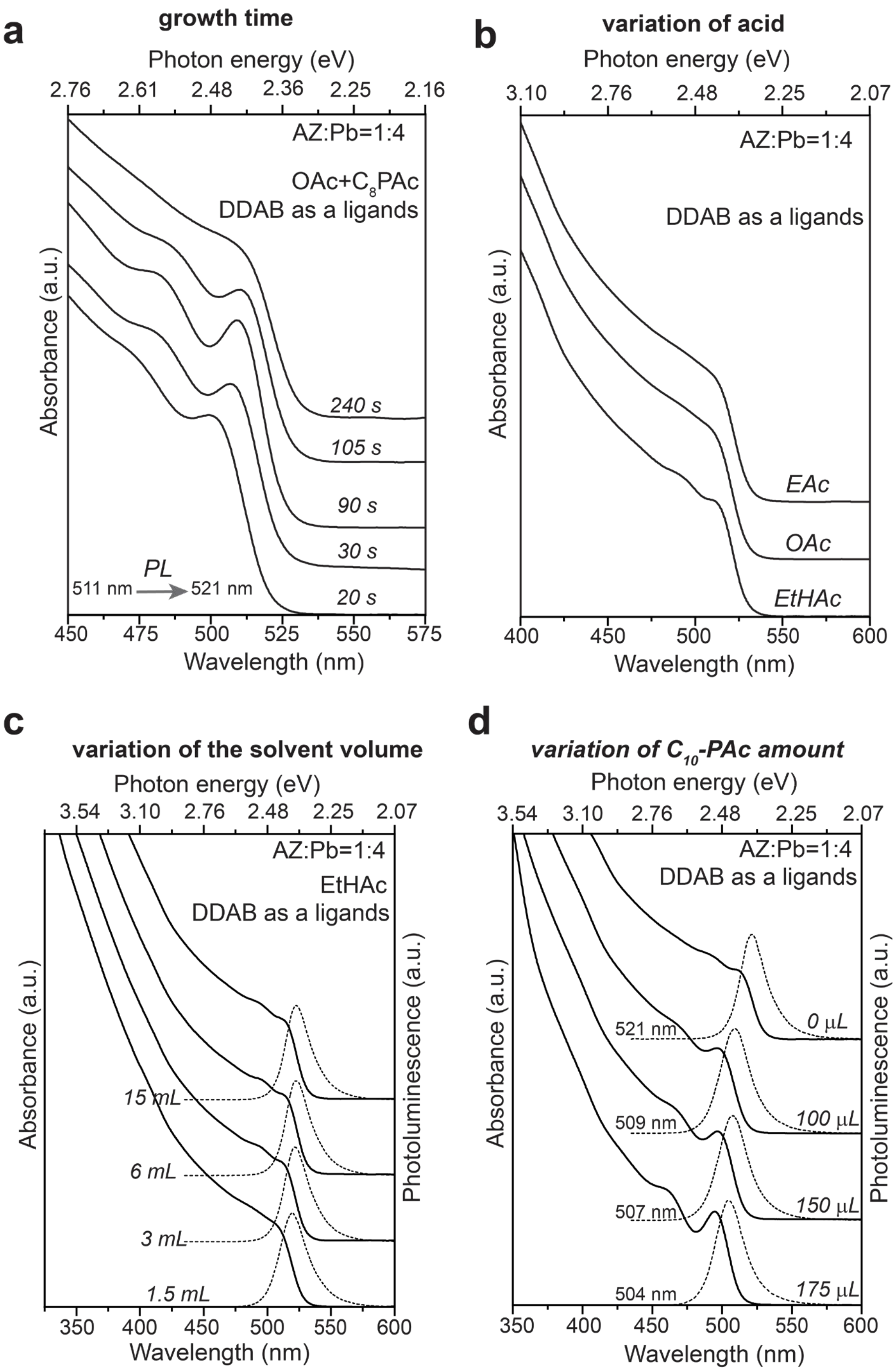

Figure S2. *Absorption spectra of AZPbBr$_3$ NCs synthesized (**a**) at various growth times and (**b**) using various acids (2-ethylhexanoic acid, oleic acid, and erucic acid) to protonate aziridine; absorption and photoluminescence spectra of AZPbBr$_3$ NCs synthesized (**c**) upon dilution showing minimal to no changes in the emission peak position and (**d**) using various amount of decylphosphonic acid showing a decrease in the size with increasing phosphonic acid concentration.*

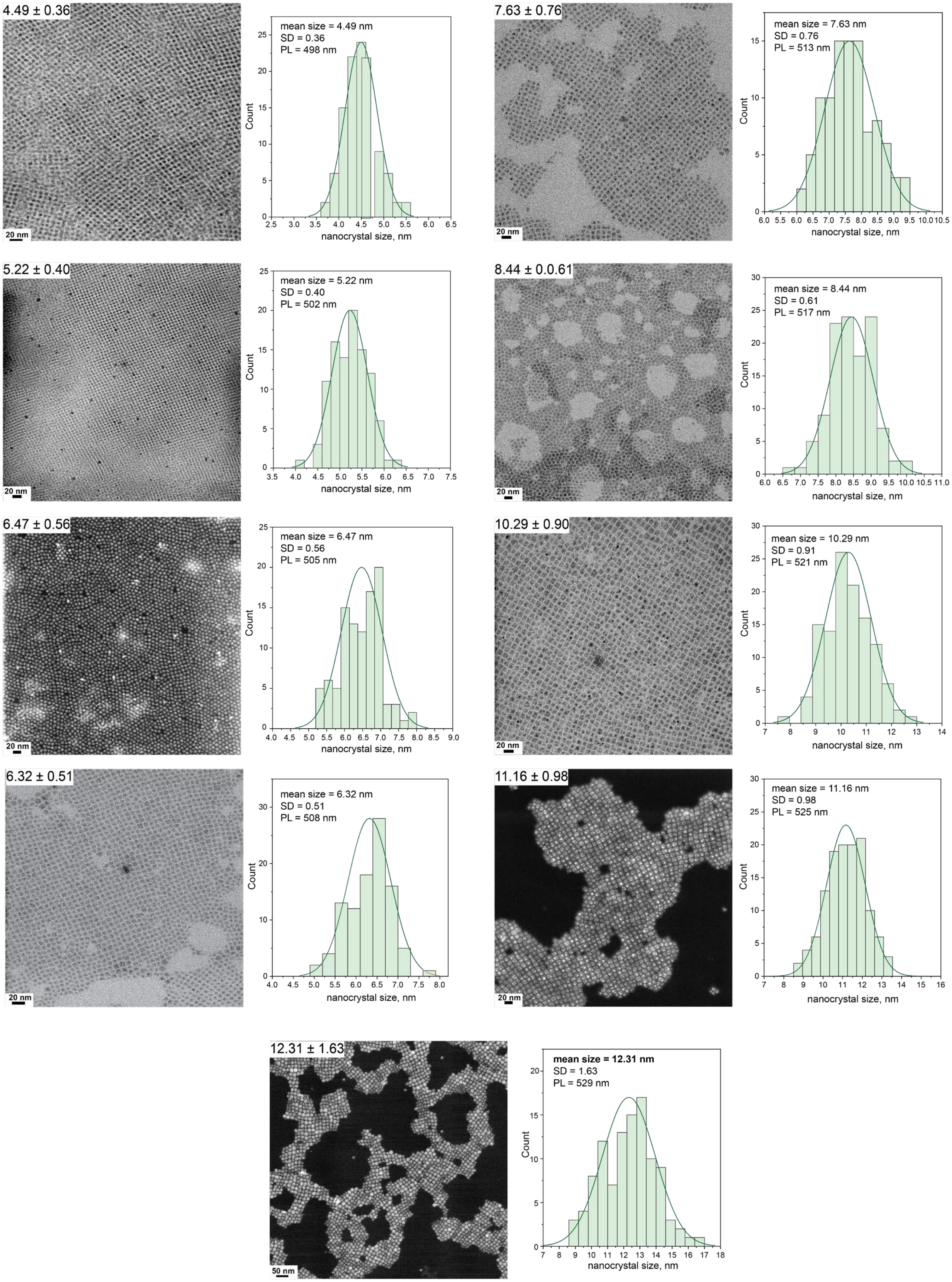

Figure S3. *Bright and dark-field STEM images of various sizes AZPbBr$_3$ NCs and corresponding size distribution histograms.*

In-situ absorption

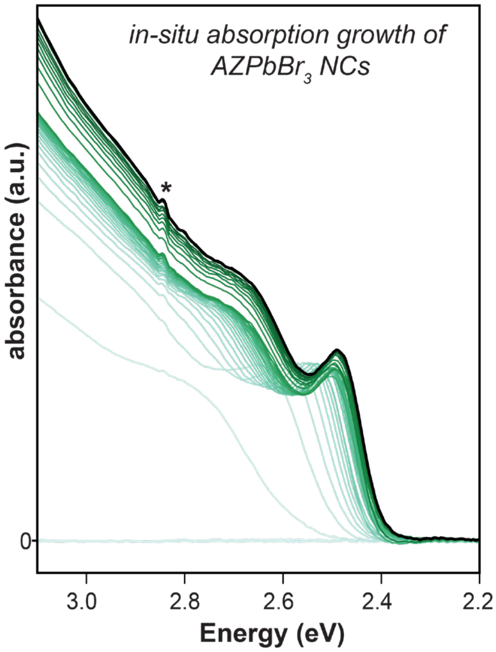

Figure S4. *In-situ recorded absorption spectra during the formation of 6-nm $AZPbBr_3$ NCs, with the black solid line being the final recorded absorption spectrum, demonstrating a clear first absorption peak.*
** - an artifact from the background room light.*

Small-angle X-ray scattering (SAXS) measurements

Data fitting. SAXS data analysis was done systematically over the available q-range for all normalized and background-subtracted samples using SASFIT 2023 software package [1–3]. The scattering profiles were fitted with a parallelepiped model of structure factor $F_P(\vec{Q})$, already implemented in the SASFit suite. For a randomly oriented ensemble of equal parallelepipeds, the scattering intensity for a random orientation parallelepiped, $P_{P,rnd}(Q)$, is defined by:

$$P_{P,rnd}(Q) = \langle F_P^2(Q) \rangle_q = \int_0^{\pi} d\theta \int_0^{2\pi} F_P^2\left(Q \begin{pmatrix} cos(\varphi) * \sin(\theta) \\ \sin(\varphi) * \sin(\theta) \\ cos(\theta) \end{pmatrix} \right) d\varphi$$

A log-normal distribution was applied to the long axis of cube-like objects to account for the samples' polydispersity when it improved the fit.

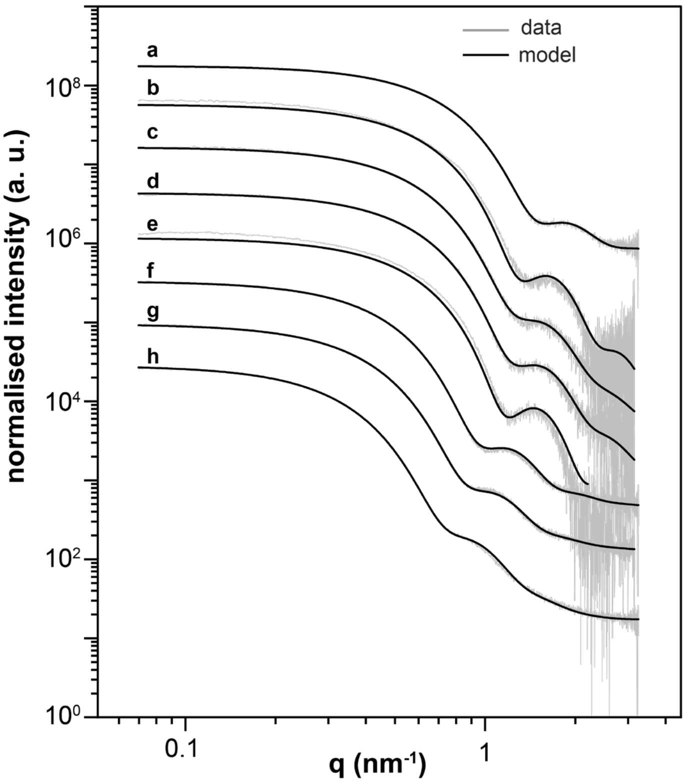

Figure S5. *SAXS traces of AZPbBr₃ NC dispersions of various sizes (gray lines) fitted via a randomly oriented parallelepiped model (black lines)*

Table S2. *Edge lengths of various sizes of AZPbBr₃ NCs (with statistical errors in the form of esd's) obtained by fitting experimental SAXS data (Figure S5) via an analytical model. Average values and maximum deviation*

Colloidal solution of $AZPbBr_3$ NCs (Fig. S5)	Short edge *SE (nm)*	Intermediate edge *IE (nm)*	Long edge *LE (nm)*	Average edge *AE (nm)*	Anisotropy *(LE-SE)/(AE)*	PL (nm)
**a**	4.48 ± 0.55	4.50 ± 0.56	4.72 ± 0.58	4.57	0.05	501
**b**	5.17 ± 0.42	5.40 ± 0.57	5.48 ± 0.57	5.35	0.06	505
**c**	5.40 ± 0.79	5.43 ± 0.79	5.44 ± 0.79	5.42	0.007	508
**d**	5.37 ± 0.70	5.70 ± 0.73	5.70 ± 0.73	5.59	0.06	512
**e**	5.93 ±0.57	5.96 ± 0.57	5.97 ± 0.57	5.95	0.007	514
**f**	6.85 ± 0.62	7.07 ± 0.91	7.42 ± 0.92	7.11	0.08	521
**g**	7.74 ± 1.18	7.76 ± 1.18	7.76 ± 1.18	7.75	0.002	523
**h**	8.94 ±1.39	9.18 ± 1.50	9.87 ± 1.47	9.33	0.09	529

Synchrotron X-ray total scattering measurements

Room temperature measurements. X-ray total scattering measurements were performed on $AZPbBr_3$ NCs as colloidal suspensions in cyclohexane and on dry samples.

These experiments were performed at two large-scale facilities with slightly different experimental conditions:

i) at the X04SA-MS beamline of the Swiss Light Source (Paul Scherrer Institute, Villigen, Switzerland),[4] by filling 0.7 mm borosilicate glass capillaries of certified composition (Hilgenberg GmbH G50) with colloidal suspensions of $AZPbBr_3$ NCs in cycloheptane (three samples). A capillary filled with a dry microcrystalline powder was also prepared as a reference. A beam energy of 22 keV was chosen, with the operational wavelength (0.56324 Å) accurately determined using a silicon powder standard (NIST 640d, a_0 = 0.543123(8) nm at 22.5°C). Scattering data were collected in the 0.1°-120° 2θ range, using a single-photon counting silicon microstrip detector (MYTHEN III).[5] Scattering from the sample holder, from empty, and from pure cycloheptane-loaded glass capillaries were independently collected under the same experimental conditions. Angular calibrations were applied to the zero angle and the x, y capillary offsets using the NIST 640d standard and locally developed procedures.

ii) at the Swiss-Norwegian beamline (BM01) of the European Synchrotron Radiation Facility (ESRF, Grenoble, France) by filling two 0.5 mm borosilicate glass capillaries of certified composition (Hilgenberg GmbH G50) with a colloidal suspension of $AZPbBr_3$ NCs and with the same sample in dry conditions, prepared by slow RT evaporation of the solvent. A beam energy of 17 keV was selected, and the scattering data were collected using a Pilatus2M detector. Scattering from the sample holder, from empty, and from pure cycloheptane-loaded glass capillaries were independently collected under the same experimental conditions. A microcrystalline LaB_6 sample was used to calibrate the experimental setup (operational wavelength, sample-to-detector distance, detector tilting), and the 2D diffraction images were azimuthally integrated to 1D intensities vs. 2θ using Bubble.[6]

For both experiments angle-dependent intensity corrections were applied to the 1D raw data to account for scattered beam attenuation due to absorption effects; sample absorption curves were determined using an X-ray tracing method[7] and by measuring the direct beam and the transmitted beam from the filled capillary at room temperature. For the empty capillary, the X-ray attenuation coefficient was computed using its nominal composition. Background and (absorption-corrected) capillary scattering contributions were subtracted from the sample signal, while the cycloheptane scattering trace was added to the $AZPbBr_3$ NC models as a blank trace, suitably rescaled by linear least squares.

Cryogenic temperature experiments. Low-temperature measurements on $AZPbBr_3$ NCs were performed at the BM01 beamline of the ESRF (Grenoble, France) in the 11-290 K temperature range by using an in-

house built He cryostat and an operational photon energy of 17 keV. The capillaries were filled with dry NCs, as detailed in the previous paragraph. The contribution to the total scattering signal coming from the cryostat with the empty capillary mounted on the support was collected and subtracted during the data reduction.

The final WAXTS data measured at different temperatures are shown in Figure S6a. Together with the scattering signal of $AZPbBr_3$ NCs, parasitic scattering from the sample holder (not properly reproduced from the collected blank trace) is visible and properly flagged with asterisks in the figure. The scattering pattern from dry NCs collected outside the cryostat at room temperature (298 K, dark red trace in Figure S8a) is shown for comparison. In all these WAXTS patterns, the 100 (Q = 1.05 $Å^{-1}$) and 200 (Q = 2.10 $Å^{-1}$) peaks appear as doublets due to atomic-scale interference of the cuboidal NCs assembled into ordered superstructures upon slowly drying, as detailed in Ref.[8] for all-inorganic perovskites NCs.

The trend of the tetragonal unit cell volume vs temperature is reported in Figure S8b, showing a non-linear behavior, approaching an almost constant value below 60 K.[9]

Debye Scattering Equation (DSE)-based WAXTS data modeling

The DSE, employed for fitting the WAXTS data reported in the main text, provides the average differential cross-section (or the powder diffraction pattern) of a randomly oriented powder (here, of identical nanocrystals) from the distribution of interatomic distances between atomic pairs[10, 11]:

$$I(Q) = \sum_{j=1}^{N} f_j(Q)^2 o_j \; + 2\sum_{j>i}^{N} f_j(Q) f_i(Q) T_j(Q) T_i(Q) o_j o_i \frac{\sin(Qd_{ij})}{(Qd_{ij})}$$

where Q = $4\pi\sin\theta/\lambda$ is the magnitude of the scattering vector, λ is the radiation wavelength, f_i is the atomic form factor of element *i*, d_{ij} is the interatomic distance between atoms *i* and *j*, *N* is the total number of atoms and *T* and *o* are the thermal atomic displacement parameter and the site occupancy factor associated to each atomic species, respectively. The first summation in the above equation includes the contributions of zero distances between one atom and itself and the second term (the interference term) the non-zero interatomic distances $d_{ij} = | r_i - r_j |$.

To compute the DSE according to the Debussy[11] strategy, atomistic models of $AZPbBr_3$ NCs of cubic shape were generated. The interatomic distances of these NCs were computed, sampled (according to a Gaussian sampling strategy, to reduce the computational times)[12], and used to feed the DSE calculation.

The DSE modeling of the WAXTS data (0.88 $Å^{-1}$ ≤ Q ≤ 13.58 $Å^{-1}$) was performed (by using both the archetypal cubic and "split cubic" models) by optimizing $AZPbBr_3$ cuboidal NC average sizes and their standard deviations (according to monovariate lognormal functions), and AZ, Pb, Br isotropic atomic displacement parameters. A comparison between the two (cubic and "split cubic") structural models is

shown in Table S7. The best fits obtained by using the "split cubic" structure[13, 14] are shown in Figure S6c for the bulk, and in Figure S8d for a selected colloidal sample, together with the histograms showing the monovariate lognormal size distribution of the cuboidal clusters (inset of Figure S6d) for the NCs.

Anisotropic prismatic models with bivariate size distributions were also tested for all the samples of $AZPbBr_3$ NCs, yielding substantially isotropic cuboidal shapes as shown in Figure 2b of the main text and in good agreement with the SAXS analysis of similar samples (Table S2).

As detailed in the main text, beyond the "split cubic" structure, a slight distortion of the cubic lattice was found for these NCs already at RT and persisting down to cryogenic temperatures. The analysis of the crystal metrics was performed by using the structureless Le Bail method with the software TOPAS [Topas-R, V3.0, 2005, Bruker AXS, Karlsruhe, Germany], and the results are shown in Figure S8e (cubic metric) and Figure S8f (tetragonal metric) for a selected colloidal sample, highlighting the improved agreement of the latter with the experimental data (R_{wp} = 2.70 vs 1.44 %, for the cubic vs tetragonal models).

Table S3. *Structural results obtained from $AZPbBr_3$ NCs WAXTS data modeling.*

**Sample**	***a*(Å)**	***Pb-Br-Pb* (°)**	**$\Delta a/a_{pow,}$ (%)**	***B*(*Pb*) (Å^2) unsplit/split**	***B*(Br) (Å^2) unsplit/split**	***GoF* unsplit/split**
**Powder**	5.99794(4)	167.75(7)	0.00%	2.41/2.40	5.46/2.12	8.12/7.23
**NCs-1**	6.0065(2)	167.2(2)	0.14%	2.18/2.24	5.35/1.76	1.35/1.31
**NCs-2**	6.0070(3)	167.1(2)	0.15%	2.18/2.24	4.94/1.36	1.31/1.28
**NCs-3**	6.0038(3)	167.0(2)	0.10%	2.08/2.13	4.93/1.32	1.26/1.24

a*-unit cell parameter* that was refined according to the cubic $Pm\overline{3}m$ crystal symmetry and a "split-cubic" structural model; $\boldsymbol{\Delta a/a_{pow}=(a-a_{pow})/a_{pow}}$ - the relative tensile strain to represent the deviation of each nanocrystalline specimen unit cell from the powder sample; ***Pb-Br-Pb*** – the Pb-Br-Pb bond angle from the split-cubic structure refined by using Rietveld Refinement and the TOPAS [Topas-R, V3.0, 2005, Bruker AXS, Karlsruhe, Germany], software; ***B*** - atomic displacement parameter (isotropic Debye-Waller factors) of Pb and Br atoms; ***GoF*** - the Goodness of fit statistical parameter of the archetypal *unsplit* (*Pb-Br-Pb = 180°*) and *split* cubic structures (with locally tilted/disordered $PbBr_6$ octahedra).

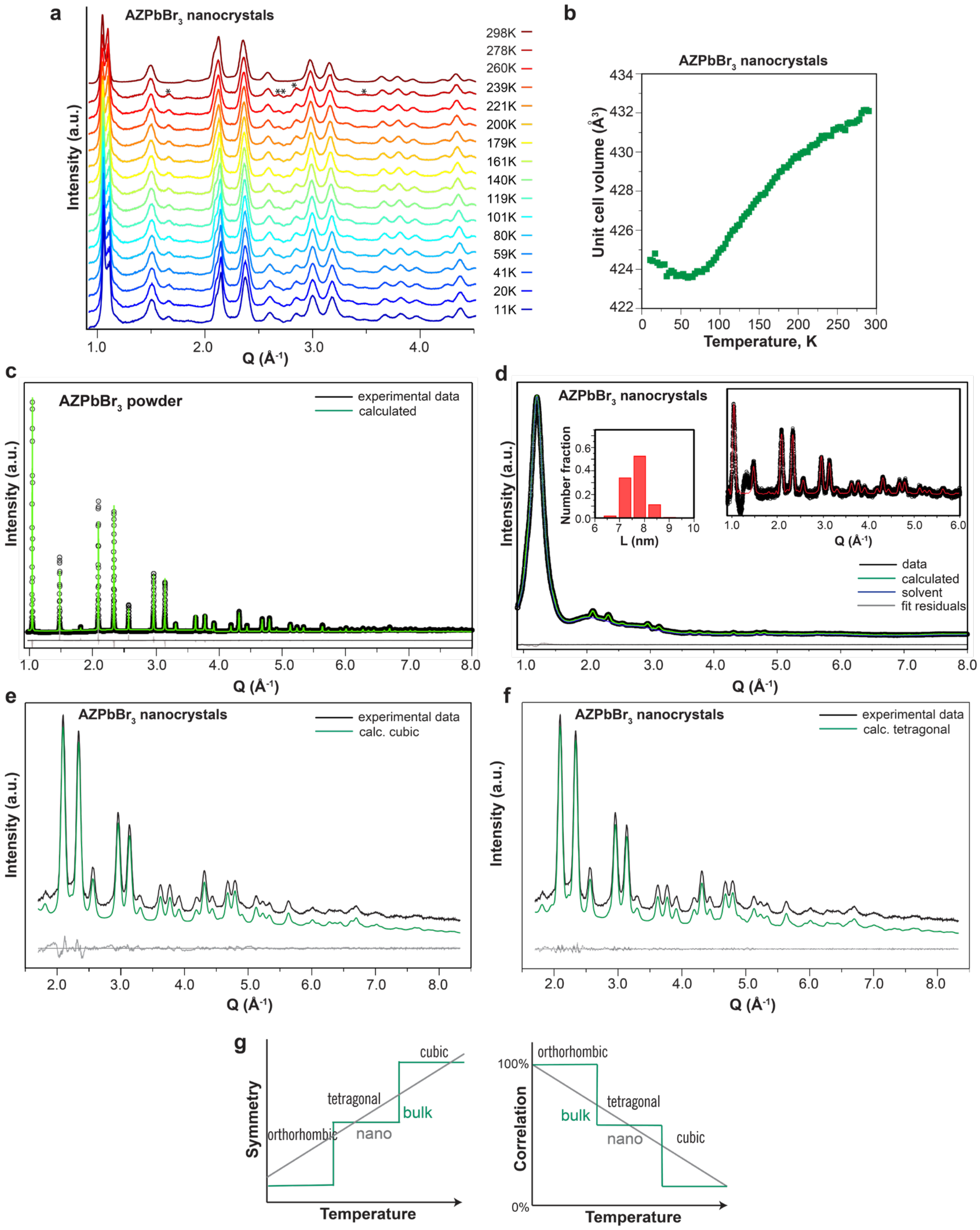

Figure S6. *(**a**) Selected variable-temperature WAXTS data of AZPbBr$_3$ dry NCs from RT down to 11K. Asterisks indicate parasitic scattering from the sample holder. (**b**) Temperature-dependent tetragonal unit-cell volume derived from AZPbBr$_3$ dry NCs data shown in a). Synchrotron WAXTS data of AZPbBr$_3$ (**c**) bulk powder and (**d**) NCs (experimental data: black dots, calculated patterns: green lines, solvent blank traces: blue lines, fit residuals: grey lines). The best fit in (c) is from Rietveld refinement, and in d) is from DSE analysis. The insets in d) show: (left) histogram plot of the refined (number-based) lognormal size distribution function (L is the edge of the cubic clusters); (right) the solvent-subtracted traces for the WAXTS-DSE best fits (red traces). Structureless Le Bail fits of one selected AZPbBr$_3$ colloidal sample obtained by using cubic (**e**) and tetragonal (**f**) metrics. The experimental data (blue curves in both e and f panels) have been subtracted by the solvent scattering independently collected in the same experimental conditions before performing this structureless analysis. (**g**) Schematic of the suggested qualitative temperature dependence of (left panel) the crystal symmetry and (right panel) the correlation among different (ideally periodic) atomic site variations (most relevant for Br ions). The depicted functional form for bulk (green) and NCs (grey) is meant as a guide to the eye only, suggesting smoother phase transitions in NCs (if detectable at all), but not conveying any quantitative features.*

We now offer an alternative description of our variable-temperature WAXTS observations. The scattering pattern, in Debye's formulation, depends on the entire set of the atom-atom distances, or, in other words, is the Fourier transform of the interatomic pair distribution function (PDF). In a perovskite, at a high enough temperature (e.g., just before melting), the atomic positions (and movements) in the different unit cells are likely poorly correlated, while the conventional crystallographic description of an average cubic lattice still holds. When the temperature is progressively lowered, correlations (of any kind) begin to rise, twisting the PDF towards showing broader and even asymmetric peaks (eventually splitting into ordered, highly correlated, low-temperature crystal phases). In reciprocal space, this turns into progressive deviations from a truly cubic pattern and may appear as parity-dependent peak displacements (as in $CsPbCl_3$ nanocrystals, see the Supporting Information of Ref.[15]), as broad superstructure peaks (the clear cubic to lower symmetry transitions of many bulk phases), or, as in the present case, as a lattice distortion which may not be related to any periodic (ordered or disordered) tetragonal lattice. Figure S6g schematically shows the different behavior of ideal bulk (green curves) and soft(er) NC matter (gray curve), where abrupt transitions are smoothed or even absent.

Computational methodology and sizing curve

The first-principles density-functional theory (DFT) calculations[16, 17] were performed using the Vienna Ab-initio Simulation Package (VASP)[18]. We used projector augmented wave (PAW)[19] pseudo-potentials within the framework of the generalized gradient approximation (GGA)[20] parametrization. The structure optimization and electronic structure calculations were performed with and without spin-orbit coupling (SOC). For reciprocal-space integration, we used a 4 × 4 × 4 Monkhorst-Pack[21] k-point grid for the cubic perovskite phase, and a 5 × 5 × 3 grid for the orthorhombic perovskite phase. The cut-off energy for a plane-wave expansion was set at 500 eV. The lattice constant and atomic positions were optimized such that residual forces acting on atoms did not exceed 1 meV/Å. Dispersion interactions were included at the DFT-D3[22] level.

Sizing-curve calculations. We fit the size-dependent band gap (obtained via the lowest-energy minimum of the second derivative of the experimental absorption spectra; see Figure 1f in the main text) with the semi-empirical "general expression" provided by Aubert et al.,[23]

$$E(d_{sphere}) = \frac{1}{2} \cdot \left[E_0 + \sqrt{E_0^2 + 8|E_0|\alpha\pi^2 \frac{R_y a_0}{\varepsilon_\infty d_0} \cdot \frac{e^{-d_{sphere}/d_0}}{\left(1 - e^{-d_{sphere}/d_0}\right)^2}} \right]$$

where E_0 is the bulk band gap, $\alpha = 0.7$, R_y is the Rydberg energy (13.606 eV), a_0 is the Bohr radius of hydrogen (0.053 nm), ε_∞ is the high-frequency dielectric constant, d_0 is the Bohr diameter, and $d_{sphere} =$

$2d_{cube}/\sqrt{3}$ is the equivalent diameter of a sphere with the same volume as our cubic QDs with edge length d_{cube}. The Bohr diameter is given by $d_0 = 8\pi \frac{\varepsilon_0 \varepsilon_\infty \hbar^2}{\mu m_0 e^2}$, where ε_0 is the vacuum permittivity, $\hbar$ the reduced Planck constant, m_0 the free electron mass, e the elementary charge, and μ the reduced effective mass of the exciton, given by $\frac{1}{\mu} = \frac{1}{m_e} + \frac{1}{m_h}$, where m_e and m_h are the electron and hole effective mass, respectively. The three material parameters are E_0, d_0, and ε_∞. In our fit implementation, the fit parameters are E_0, μ, and ε_∞. E_0 and μ are free fit parameters, while ε_∞=8.25 is fixed to an estimate obtained via DFT calculations: due to our calculated directionally-averaged zero-frequency dielectric constant being 13% higher in $AZPbBr_3$ than in $CsPbBr_3$ (see the PBE+vdW+SOC values in Table S5), we set ε_∞=8.25 for $AZPbBr_3$, 13% higher than the experimental value of 7.3 reported for $CsPbBr_3$ by Yang et al.[24] The fit to the size-dependent band gap for both the SAXS and the TEM data set are shown in Figure 1f of the main text and the associated fit parameters are summarized in Table S6.

Table S4: *Calculated effective masses of electrons (m_e) and holes (m_h) along various symmetry lines for orthorhombic $AZPbBr_3$ and orthorhombic $CsPbBr_3$, at the PBE+vdW+SOC level of theory. m_o is the rest mass of an electron.*

	m_h/m_o			m_e/m_o		
Symmetry line	*Γ-X*	*Γ -S*	*Γ -Z*	*Γ –X*	*Γ -S*	*Γ -Z*
**AzPbBr₃**	0.168	0.175	0.166	0.176	0.168	0.153
**CsPbBr₃**	0.234	0.239	0.190	0.229	0.239	0.187

Table S5: *The components of the static dielectric constant ($\varepsilon_1(0)$) for ground-state structures of $AZPbBr_3$ and $CsPbBr_3$ in the orthorhombic phase, calculated using an independent-particle approximation.*

	**PBE+vdW**			**PBE+vdW+SOC**		
Component	*X*	*Y*	*Z*	*X*	*Y*	*Z*
**$AZPbBr_3$**	4.7345	4.7898	4.9714	5.1419	5.2116	5.4678
**$CsPbBr_3$**	4.5991	4.6697	4.7842	4.5825	4.6531	4.7655

Table S6: *Fit parameters obtained from the fit to the $AZPbBr_3$ size-dependent band gap displayed in Figure 1f of the main text according to T. Aubert et al.*[23]

**Method for size determination**	**Bulk band gap E_0**	**Reduced mass μ**	**Dielectric constant ε_∞**	**Bohr diameter d_0**
**SAXS**	2.346 eV (free)	0.173 (free)	8.25 (fixed, estimated via DFT)	5.05 nm
**TEM**	2.381 eV (free)	0.183 (free)	8.25 (fixed, estimated via DFT)	4.76 nm

Crystal structures and energetics of $AZPbBr_3$

At first, DFT was used to identify the most energetically stable structure of $AZPbBr_3$. Table S6 reports the calculated total energy per formula unit of $AzPbBr_3$ in either cubic or orthorhombic crystal phase, at three different levels of theory. A simple PBE calculation suggests that the cubic phase may be more stable than the orthorhombic phase, by 12 meV energy (Table S7). However, as van der Waals (vdW) interactions were previously found to be significant for the polymorphism[19], we next implemented also PBE+vdW calculations. This approach showed that the orthorhombic phase is more stable than the cubic phase, by about 41 meV. This energetic ordering of crystal structures is consistent with the expectation that the larger degrees of freedom of the orthorhombic phase should enable an equal or lower energy than for the cubic phase. Additionally, it highlights the importance of vdW interactions in DFT calculations for ionic solids, especially for materials containing organic components such as $AZPbBr_3$. Finally, since the Pb atom is present in $AZPbBr_3$, we also considered spin-orbit coupling (SOC). The PBE+vdW+SOC approach confirms the orthorhombic phase as the most energetically stable phase at 0 K, while the only 45 meV higher energy of the cubic phase (similar as in the PBE+vdW calculations) points toward the possibility of some polymorphism in $AZPbBr_3$. In Table S8, we have reported the optimized cell parameters and equilibrium volumes of $AzPbBr_3$ in both the cubic and the orthorhombic phase.

Table S7. *Calculated total energies per formula unit of $AZPbBr_3$ in the cubic and orthorhombic phase.*

Phase		PBE			PBE+vdW			PBE+vdW+SOC		
			Energy (eV)/fu	E_g (eV)		Energy (eV)/fu	E_g (eV)		Energy (eV)/fu	E_g (eV)
Cubic			-60.3850	1.881		-61.7218	1.693		-61.9616	0.755
Orthorhombic			-60.3730	2.007		-61.7633	1.891		-62.0067	0.871

Table S8. *Optimized cell parameters and volume of $AZPbBr_3$ in the cubic and orthorhombic phase.*

Phase	PBE			PBE+vdw			PBE+vdw+SOC		
	a/b/c (Å)	Angles (°)	Vol (Å³)	a/b/c (Å)	Angles (°)	Vol (Å³)	a/b/c (Å)	Angles (°)	Vol (Å³)
Cubic	6.1110<br>6.0587<br>6.2202	90.00<br>89.94<br>90.00	230.298	5.9657<br>5.9689<br>6.0801	90.00<br>90.00<br>90.00	216.873	5.9630<br>5.9667<br>6.0856	90.00<br>90.00<br>90.00	216.739
Orthorhombic	8.4812<br>8.7199<br>12.1732	89.19<br>90.00<br>90.00	900.196	8.2692<br>8.5275<br>11.9703	90.00<br>90.00<br>90.00	844.100	8.2677<br>8.5205<br>11.9584	90.00<br>90.00<br>90.00	842.421

As a final remark, we note that our approach for the "cubic" models included a non-kosher relaxation of the a = b = c axial lengths equalities, which resulted in the a ~ b < c order [see PBE+vdw (+SOC) columns].

Possibly, this computational result suggests a lattice distortion from pristine "cubicity" towards a tetragonal metrics (as experimentally evidenced for the dry NC samples, where the √2:√2:1 axial length relationship (after transformation) approximately holds. Worthy of note, also the computed c/a ratio (of ca. 1.019) appears to be well in line with the 1.013 value experimentally determined in the 100-290K range.

Electronic structure of $AZPbBr_3$

Figure S7 illustrates the band structure of $AZPbBr_3$ in the orthorhombic phase, computed at the PBE+vdW+SOC level of theory, *i.e.*, within the PBE framework and including both van-der-Waals (vdW) interactions and spin-orbit coupling (SOC). $AZPbBr_3$ is a direct band-gap semiconductor, with its fundamental band gap positioned at the Γ-point of the first Billouin zone. Inclusion of SOC strongly modifies the electronic band structures of $AZPbBr_3$, with a reduced band gap of about 0.87 eV and a sizable splitting of bands,. The splitting is especially pronounced in the conduction band, particularly along the Y-Γ-X line (inset in Figure S7). This phenomenon might be attributed to the fact that the conduction band originates from Pb-p orbitals while the valence band is dominated by Br-p orbitals, as elaborated below. An important point is that SOC maintains the direct band gap feature of $AZPbBr_3$ at the Γ-point. Figure S8 shows the projected and total density of states of $AZPbBr_3$ with SOC included. We found that the valence-band maximum (VBM) is dominated by Br-p orbitals with a small admixture of Pb-s orbitals. On the other hand, the conduction-band minimum (CBM) mainly originates from Pb-p orbitals. N-p and C-p orbitals contribute only deeper inside the valence band, below an energy of about -4.5 eV. As shown in Table S4, both electrons and holes exhibit small effective masses of $< 0.2\ m_o$, slightly smaller than in $CsPbBr_3$.

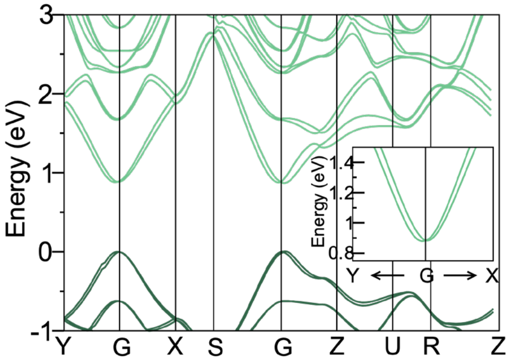

Figure S7. *The electronic band structure of orthorhombic $AZPbBr_3$ at the PBE+vdW+SOC level of theory. The inset shows the band splitting along the Y-Γ-X symmetry line.*

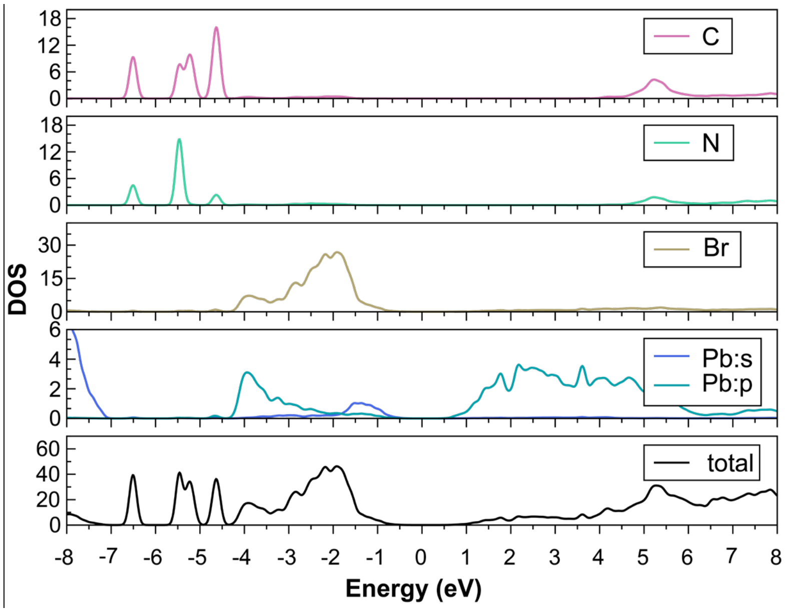

Figure S8. *The total and projected density of states of AZPbBr$_3$ at the PBE+vdW+SOC level of theory. The energy axis is aligned such that the Fermi level is at 0 eV.*

NMR measurements

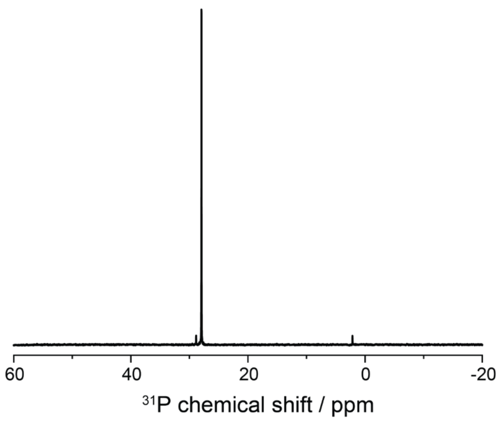

Figure 9. *Solution ^{31}P NMR of octylphosphonic acid (C_8PAc) in DMSO-d_6/toluene-d_8 (1:1). A main signal at 28 ppm is observed with two minor impurities at 2 and 29 ppm.*

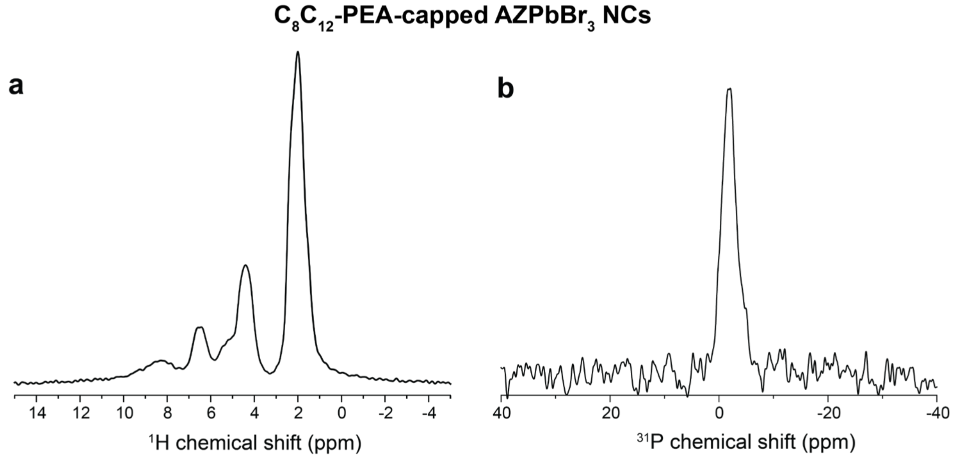

Figure S10. *Solid-state (a) ^1H and (b) ^{31}P NMR spectra of C_8C_{12}-PEA-capped $AZPbBr_3$ NCs.*

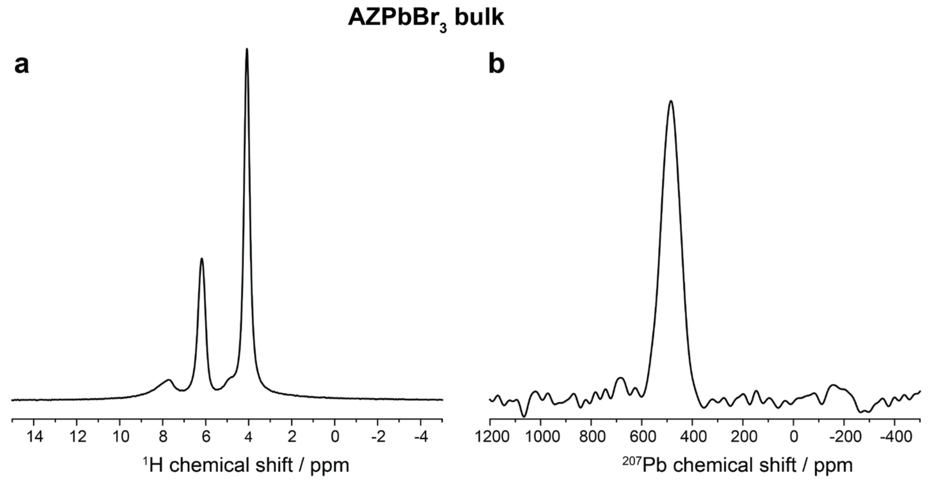

Figure S11. *Solid-state (a) ^1H and (b) ^{207}Pb NMR spectra of bulk $AZPbBr_3$.*

Powder X-ray diffraction

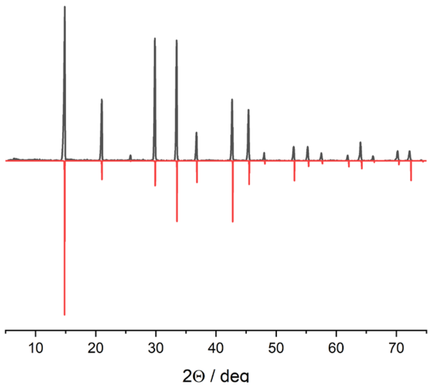

Figure S12. *Powder XRD pattern of the bulk $AZPbBr_3$ (black) and the calculated pattern of the crystal structure from the literature (red).*[3] *The slight mismatch between the intensity ratios of the black (experimental data) and red (calculated data) is attributed to morphological effects (preferred orientation), here not modeled.*

Raman spectroscopy

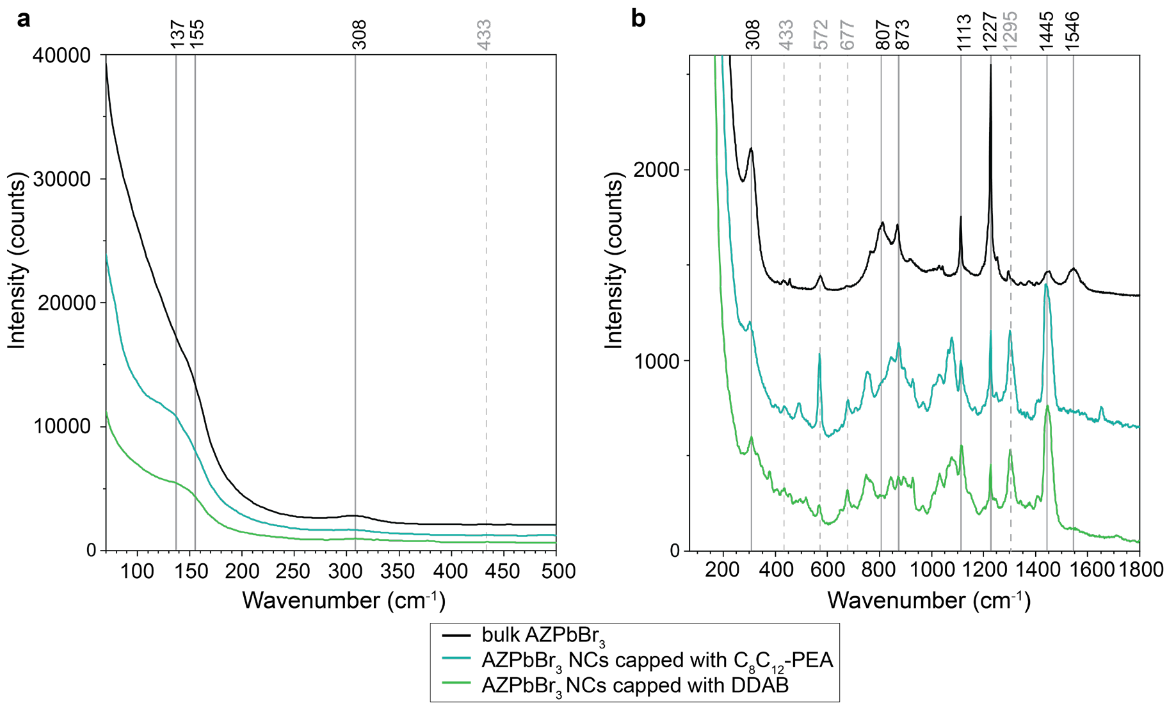

Figure S13. *Raman spectra in the range of (a) 70-500 cm^{-1} and (b) 70-1800 cm^{-1} of DDAB-capped AZPbBr$_3$ NCs (green line), C$_8$C$_{12}$-PEA-capped AZPbBr$_3$ NCs (blue line), and AZPbBr$_3$ bulk powder (black line), respectively.*

**NCs**	**bulk**	**Ref.**[25]	**Proposed assignment**
137	155	132	Pb-Br stretch
**307**	**308**	**308**	**AZ cage mode**
433	*433*	*429*	*Degradation product*
569	*572*	*573*	*Degradation product*
678	*677*	*677*	*Degradation product*
**807**	**807**	**802**	**Ring deformation**
**874**	**871**	**871**	**Ring deformation**
**1113**	**1113**	**1113**	**ω(CH_2)**
**1227**	**1227**	**1228**	**Ring stretch**
1295	*1302*	*1317*	*Degradation product*
**~1445**	**~1445**	**1440-1453**	**δ(CH_2), 2 peaks**
**weak**	**1546**	**1547**	**δ(NH_2)**

Table S9. *Raman scattering peak locations (wavenumbers, in cm^{-1}) observed for AZPbBr$_3$ NCs and AZPbBr$_3$ bulk material together with the proposed assignments, following Ref.* [25]

Single-dot spectroscopy

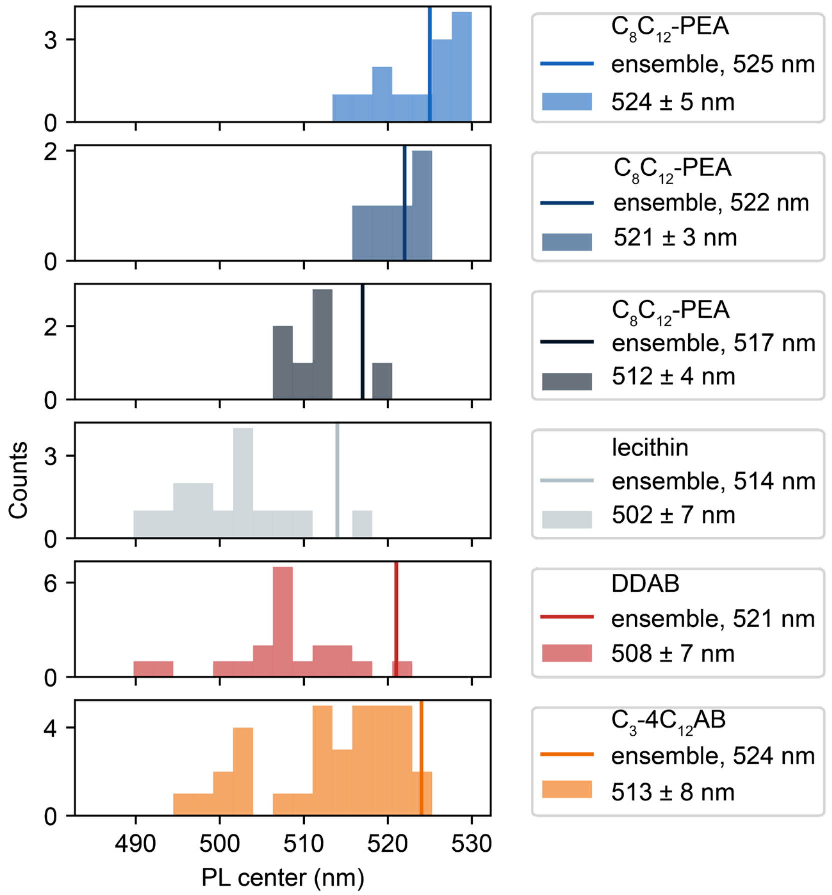

Figure S14. *Static blueshift of single* $AZPbBr_3$ *NCs***.** *Histogram of PL peak centers of the single NCs (bars) and corresponding ensemble PL peak centers (line) characterizing static blueshift induced by dilution during sample preparation. The numbers after the "ensemble" indicate the ensemble PL central wavelength of the respective sample.*

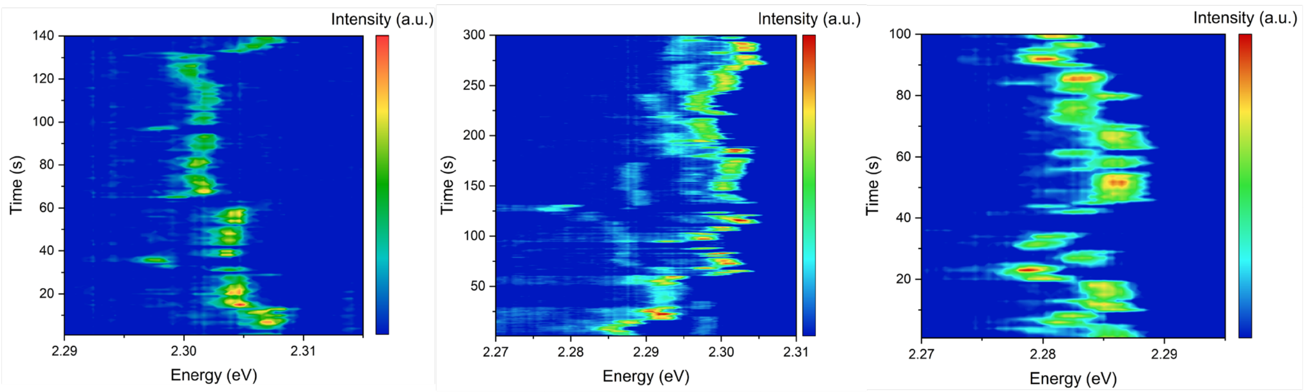

Figure S15. *Typical spectral stability of single* $AZPbBr_3$ *NCs capped with lecithin ligands. Such NCs are generally less stable than the ones capped with DDAB or* C_8C_{12}*-PEA. They show typically strong spectral diffusion (~ 10 meV), rendering the evaluation of exciton fine-structure splitting unfeasible. The time series (1s integration time) displayed here are acquired with a 300g/mm grating.*

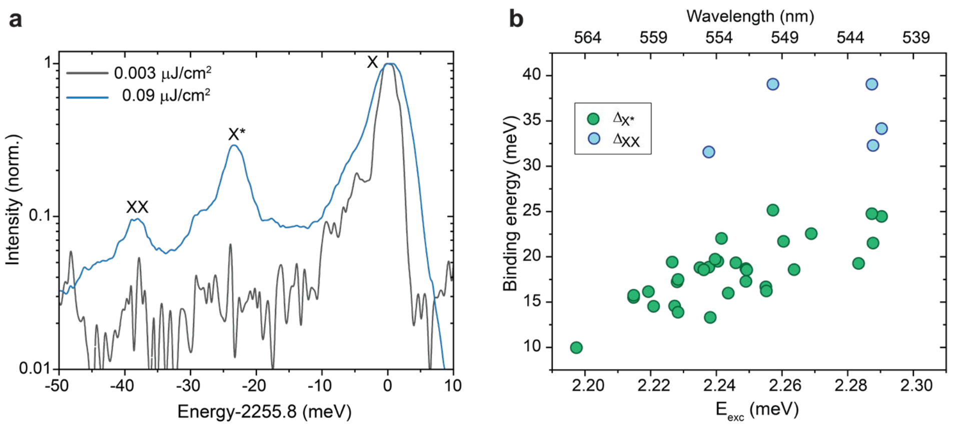

Figure S16. *The binding energy of trion (Δ_{X^*}) and biexciton (Δ_{XX}) of single AZPbBr$_3$ NCs at 4 K.* ***(a)*** *Spectra of a single AZPbBr$_3$ NC under excitation fluences of 0.003 $\mu J/cm^2$ (dark curve) and 0.09 $\mu J/cm^2$ (blue curve). The spectra are referenced with respect to the single-exciton (X) energy (2.2558 eV). At high excitation fluence (0.09 $\mu J/cm^2$), trion and biexciton emissions emerge in the lower-energy sidebands of the exciton emission.* ***(b)*** *Binding energies of trion (Δ_{X^*}) and biexciton (Δ_{XX}) as a function of the exciton energy E_{exc}. Δ_{X^*} increases from 10 to 25 meV for NCs with exciton energies from 2.20 to 2.29 eV. Δ_{XX} increases from 30 meV to 40 meV for NCs with exciton energies from 2.23 to 2.29 eV.*

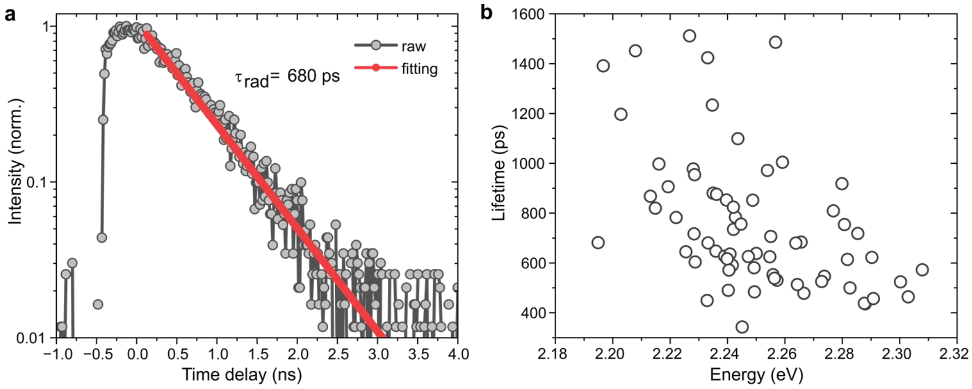

Figure S17. *Lifetime of single AZPbBr$_3$ NCs at 4 K.* ***(a)*** *Representative time-resolved PL trace of a single AZPbBr$_3$ NC, exhibiting a mono-exponential decay with a lifetime of around 680 ps (marked as the filled circle in* ***(b)****).* ***(b)*** *Lifetimes of several single AZPbBr$_3$ NCs as a function of their exciton energies.*

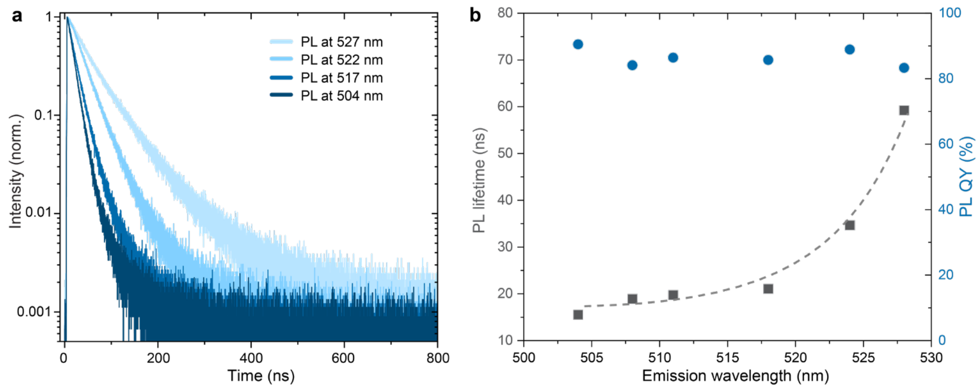

Figure S18. *Size-dependent exciton lifetime in AZPbBr$_3$ NCs.* ***(a)*** *Exemplary time-resolved PL traces obtained at room temperature by exciting the colloidal solutions with a 355 nm pulsed laser (repetition rate of 1 MHz).* ***(b)*** *Extracted exciton lifetime (grey squared data points) for samples with different emission wavelength together with the measured PL QY (blue circle data points) for all the here studied samples.*

References

1. Morad, V.; Stelmakh, A.; Svyrydenko, M.; et al. Designer Phospholipid Capping Ligands for Soft Metal Halide Nanocrystals. *Nature* **2023**, DOI: 10.1038/s41586-023-06932-6.
2. Ginterseder, M.; Sun, W.; Shcherbakov-Wu, W.; McIsaac, A. R.; Berkinsky, D. B.; Kaplan, A. E. K.; Wang, L.; Krajewska, C.; Šverko, T.; Perkinson, C. F.; Utzat, H.; Tisdale, W. A.; Van Voorhis, T.; Bawendi, M. G. Lead Halide Perovskite Nanocrystals with Low Inhomogeneous Broadening and High Coherent Fraction through Dicationic Ligand Engineering. *Nano Lett.* **2023,** *23*, 1128-1134.
3. Petrosova, H. R.; Kucheriv, O. I.; Shova, S.; Gural'skiy, I. y. A. Aziridinium Cation Templating 3D Lead Halide Hybrid Perovskites. *Chem. Comm.* **2022,** *58*, 5745-5748.
4. Willmott, P. R.; Meister, D.; Leake, S. J.; Lange, M.; Bergamaschi, A.; al., e. The Materials Science Beamline Upgrade at the Swiss Light Source. *J. Synchrotron Rad.* **2013,** *20*, 667-682.
5. Bergamaschi, A.; Cervellino, A.; Dinapoli, R.; Gozzo, F.; Henrich, B.; Johnson, I.; Kraft, P.; Mozzanica, A.; Schmitt, B.; Shi, X. The MYTHEN Detector for X-ray Powder Diffraction Experiments at the Swiss Light Source. *J. Synchrotron Rad.* **2010,** *17*, 653-668.
6. Dyadkin, V.; Pattison, P.; Dmitriev, V.; Chernyshov, D. A New Multipurpose Diffractometer PILATUS@SNBL. *J. Synchrotron Rad.* **2016,** *23*, 825-829.
7. Bowden, M.; Ryan, M. Absorption Correction for Cylindrical and Annular Specimens and Their Containers or Supports. *J. Appl. Crystallogr.* **2010,** *43*, 693-698.
8. Bertolotti, F.; Vivani, A.; Ferri, F.; Anzini, P.; Cervellino, A.; Bodnarchuk, M. I.; Nedelcu, G.; Bernasconi, C.; Kovalenko, M. V.; Masciocchi, N.; Guagliardi, A. Size Segregation and Atomic Structural Coherence in Spontaneous Assemblies of Colloidal Cesium Lead Halide Nanocrystals. *Chem. Mater.* **2022,** *34*, 594-608.
9. Kroll, H.; Kirfel, A.; Heinemann, R.; Barbier, B. Volume Thermal Expansion and Related Thermophysical Parameters in the Mg,Fe Olivine Solid-Solution Series. *Eur. J. Mineral.* **2012,** *24*, 935-956.
10. Debye, P. Zerstreuung von Röntgenstrahlen. *Ann. Phys.* **1915,** *351*, 809-823.
11. Cervellino, A.; Frison, R.; Bertolotti, F.; Guagliardi, A. DEBUSSY 2.0: the New Release of a Debye User System for Nanocrystalline and/or Disordered Materials. *J. Appl. Crystallogr.* **2015,** *48*, 2026-2032.
12. Cervellino, A.; Giannini, C.; Guagliardi, A. On the Efficient Evaluation of Fourier Patterns for Nanoparticles and Clusters. *J. Comput. Chem.* **2006,** *27*, 995-1008.
13. Protesescu, L.; Yakunin, S.; Bodnarchuk, M. I.; Bertolotti, F.; Masciocchi, N.; Guagliardi, A.; Kovalenko, M. V. Monodisperse Formamidinium Lead Bromide Nanocrystals with Bright and Stable Green Photoluminescence. *J. Am. Chem. Soc.* **2016,** *138*, 14202-14205.
14. Hanusch, F. C.; Wiesenmayer, E.; Mankel, E.; Binek, A.; Angloher, P.; Fraunhofer, C.; Giesbrecht, N.; Feckl, J. M.; Jaegermann, W.; Johrendt, D.; Bein, T.; Docampo, P. Efficient Planar Heterojunction Perovskite Solar Cells Based on Formamidinium Lead Bromide. *J. Phys. Chem. Lett.* **2014,** *5*, 2791-2795.
15. Bertolotti, F.; Protesescu, L.; Kovalenko, M. V.; Yakunin, S.; Cervellino, A.; Billinge, S. J. L.; Terban, M. W.; Pedersen, J. S.; Masciocchi, N.; Guagliardi, A. Coherent Nanotwins and Dynamic Disorder in Cesium Lead Halide Perovskite Nanocrystals. *ACS Nano* **2017,** *11*, 3819-3831.
16. Hohenberg, P.; Kohn, W. Inhomogeneous Electron Gas. *Phys. Rev.* **1964,** *136*, B864-B871.
17. Kohn, W.; Sham, L. J. Self-Consistent Equations Including Exchange and Correlation Effects. *Phys. Rev.* **1965,** *140*, A1133-A1138.
18. Kresse, G.; Furthmüller, J. Efficient Iterative Schemes for ab initio Total-Energy Calculations Using a Plane-Wave Basis Set. *Phys. Rev. B* **1996,** *54*, 11169-11186.
19. Blöchl, P. E. Projector Augmented-Wave Method. *Phys. Rev. B* **1994,** *50*, 17953-17979.

20. Perdew, J. P.; Burke, K.; Ernzerhof, M. Generalized Gradient Approximation Made Simple. *Phys. Rev. Lett.* **1996,** *77*, 3865-3868.
21. Monkhorst, H. J.; Pack, J. D. Special Points for Brillouin-Zone Integrations. *Phys. Rev. B* **1976,** *13*, 5188-5192.
22. Grimme, S.; Antony, J.; Ehrlich, S.; Krieg, H. A Consistent and Accurate ab initio Parametrization of Density Functional Dispersion Correction (DFT-D) for the 94 Elements H-Pu. *J. Chem. Phys.* **2010,** *132*, 154104.
23. Aubert, T.; Golovatenko, A. A.; Samoli, M.; Lermusiaux, L.; Zinn, T.; Abécassis, B.; Rodina, A. V.; Hens, Z. General Expression for the Size-Dependent Optical Properties of Quantum Dots. *Nano Lett.* **2022,** *22*, 1778-1785.
24. Yang, Z.; Surrente, A.; Galkowski, K.; Miyata, A.; Portugall, O.; Sutton, R. J.; Haghighirad, A. A.; Snaith, H. J.; Maude, D. K.; Plochocka, P.; Nicholas, R. J. Impact of the Halide Cage on the Electronic Properties of Fully Inorganic Cesium Lead Halide Perovskites. *ACS Energy Lett.* **2017,** *2,* 1621-1627.
25. Stefańska, D.; Ptak, M.; Mączka, M. Synthesis, Photoluminescence and Vibrational Properties of Aziridinium Lead Halide Perovskites. *Molecules* **2022,** *27*, 7949.
